# Vps34/PI3KC3 deletion in kidney proximal tubules impairs apical trafficking and blocks autophagic flux, causing a Fanconi-like syndrome and renal insufficiency

**DOI:** 10.1038/s41598-018-32389-z

**Published:** 2018-09-20

**Authors:** Giuseppina Grieco, Virginie Janssens, Héloïse P. Gaide Chevronnay, Francisca N’Kuli, Patrick Van Der Smissen, Tongsong Wang, Jingdong Shan, Seppo Vainio, Benoit Bilanges, François Jouret, Bart Vanhaesebroeck, Christophe E. Pierreux, Pierre J. Courtoy

**Affiliations:** 1 Cell Biology Unit, de Duve Institute, Université catholique de Louvain, Brussels, Belgium; 2 Laboratory of Developmental Biology, Oulu Center for Cell-Matrix Research, Biocenter Oulu and Faculty of Biochemistry and Molecular Medicine, University of Oulu, Oulu, Finland; 3 UCL Cancer Institute, University College London, 72 Huntley Street, London, WC1E 6DD UK; 4 Groupe Interdisciplinaire de Génoprotéomique Appliquée (GIGA), Cardiovascular Sciences, University of Liège, Liège, Belgium

## Abstract

Kidney proximal tubular cells (PTCs) are highly specialized for ultrafiltrate reabsorption and serve as paradigm of apical epithelial differentiation. Vps34/PI3-kinase type III (PI3KC3) regulates endosomal dynamics, macroautophagy and lysosomal function. However, its *in vivo* role in PTCs has not been evaluated. Conditional deletion of Vps34/PI3KC3 in PTCs by Pax8-Cre resulted in early (P7) PTC dysfunction, manifested by Fanconi-like syndrome, followed by kidney failure (P14) and death. By confocal microscopy, Vps34^∆/∆^ PTCs showed preserved apico-basal specification (brush border, NHERF-1 *versus* Na^+^/K^+^-ATPase, ankyrin-G) but basal redistribution of late-endosomes/lysosomes (LAMP-1) and mis-localization to lysosomes of apical recycling endocytic receptors (megalin, cubilin) and apical non-recycling solute carriers (NaPi-IIa, SGLT-2). Defective endocytosis was confirmed by Texas-red-ovalbumin tracing and reduced albumin content. Disruption of Rab-11 and perinuclear galectin-3 compartments suggested mechanistic clues for defective receptor recycling and apical biosynthetic trafficking. p62-dependent autophagy was triggered yet abortive (p62 co-localization with LC3 but not LAMP-1) and PTCs became vacuolated. Impaired lysosomal positioning and blocked autophagy are known causes of cell stress. Thus, early trafficking defects show that Vps34 is a key *in vivo* component of molecular machineries governing apical vesicular trafficking, thus absorptive function in PTCs. Functional defects underline the essential role of Vps34 for PTC homeostasis and kidney survival.

## Introduction

Kidney proximal tubular cells (PTCs) show exquisite structural differentiation that supports their huge reabsorption activity and serve as paradigm for apical polarity^1^. Efficient reabsorption of water and solutes from the ultrafiltrate is ensured by abundance of channels and transporters (NaPi-IIa for phosphate, SGLT-2 for glucose) at the highly developed apical brush border membrane. These transporters are anchored by ezrin and by multi-PDZ scaffold proteins including Na^+^/H^+^-exchanger regulatory factor-1 (NHERF-1)^2–5^. Transepithelial transport is achieved by complementary sets of channels and transporters inserted into infolded basolateral membrane and complemented by Na^+^/K^+^-ATPase as primary transport driving force, itself stabilized by ankyrin-G scaffold. Full reabsorption of ultrafiltrated proteins by apical receptor-mediated endocytosis is no less remarkable, considering the brief contact of PTCs with rapidly passing urine. Full reabsorption is made possible by high expression of multiligand tandem receptors, megalin and cubilin^6^, combined with extremely fast apical endocytic recycling rate^7^. Megalin trafficks via sequential endosomal compartments that define the fast, Rab11-dependent apical recycling in epithelial cells^8^. Megalin/cubilin ligands dissociate in endosomes and are selectively transferred to lysosomes for degradation. Albumin internalization by the FcRn receptor follows a parallel pathway leading to transcytosis of intact protein^9^.

Establishment and maintenance of differentiated PTC polarity depend on the orchestrated action of primary polarity clues; specification of junctional, apical, lateral and basal membrane domains; recognition of protein targeting/retention motives; and further membrane domain expansion combined with regulation of sequential endosomal sorting. Biosynthetic apical sorting of membrane proteins can occur at the trans-Golgi complex via two distinct sets of vesicles, based on either inclusion of GPI-anchored proteins into rafts, or raft-independent recognition of complex N-glycans by galectin-3^10^. Galectin-3 has recently received further recognition as luminal marker of damaged endolysosomes^11^. Endosomes play not only a key role in endocytic trafficking but also for the biosynthetic control of plasma membrane composition^8,12–14^. Mutations affecting vesicular trafficking have severe consequences in kidney function^15^. In PTCs, impaired apical trafficking due to defective recycling causes a generalized reabsorption deficit, known as kidney Fanconi syndrome, in Dent’s disease^16,17^, Lowe disease^18^ and possibly cystinosis^19^.

The vacuolar protein sorting-34 (Vps34) gene product was discovered in budding yeast as component of the machinery governing traffic to the vacuole, the equivalent of lysosomes, and is the ortholog and sole member of the class III subgroup of PI3K in multicellular organisms (PI3KC3)^20^. Vps34 lipid-kinase can only use phosphatidylinositol as substrate to produce phosphatidylinositol 3-phosphate (PtdIns3P, or PI3P) and is considered its main producer. However, other pathways e.g. via class II PI3K can also produce PI3P^21–23^. Alternatively, PI3P can be generated at endosomes by dephosphorylation of diphosphoinositides by PdIns(3,5P)_2_ phosphatases^24^.

Vps34 functions in two types of multiprotein complexes, regulating endocytic trafficking and autophagy, respectively^20^. A key role of Vps34 in endocytic trafficking was originally identified from *in vitro* fusion assays, as catalyzing recruitment of the early endosomal effectors, EEA1 and Rab5^25^, but Vps34 acts at multiple endocytic trafficking levels, including late endosomes/lysosomes^26,27^. Vps34 impact on endosomal signaling^28^ may explain its requirement for kidney compensatory growth after unilateral nephrectomy^29^, thus potentially for kidney development.

Vps34 is also known as a core component of the macroautophagy initiation machinery^30^. Macroautophagy delivers cytoplasmic contents, including mitochondria (mitophagy), to lysosomes for degradation via double-membrane structures called autophagosomes which depend on phospholipid recruitment via LC3. Selective autophagy of aggregated ubiquitinylated cytosolic proteins is achieved by p62-dependent recruitment of LC3^31^. Chaperone-mediated autophagy (CMA) is a distinct process whereby individual HSP-chaperoned proteins are translocated across the lysosomal membrane by a dedicated transporter, LAMP-2A^32^. After cell stress or injury, activation of CMA normally follows macroautophagy but CMA can compensate defective macroautophagy^33^. CMA is strongly upregulated in renal epithelial cells by EGF and TGFβ-1, which thereby govern the level of transcription factors such as Pax-2^34^, thus impacting on tissue differentiation.

Since full gene Vps34 KO causes early embryonic lethality^35^, its role *in vivo* has been addressed by conditional inactivation in various lineages: myocytes^36^, cardiomyocytes^37^, hepatocytes^37^, T-cells^38^, neurons^39^ and kidney podocytes^40–42^. In podocytes, conditional Vps34 deletion caused podocyte dysfunction after ∼3 weeks and death after 3–9 weeks^40,41^. Interestingly, although also impacting autophagy, deleterious effects of Vps34 deletion were specifically ascribed to altered endocytosis^42^. The role of Vps34 in PTCs *in vivo* has not been studied. In the polarized OK cell lines as a PTC model^43^, we reported that acute pharmacological inhibition of Vps34 did not impair apical endocytosis *per se* (megalin internalization) but prevented megalin recycling from endosomes, without affecting lysosomes^44^. Remarkably, inhibitor withdrawal unleashed a burst of endosomal tubules, indicating that Vps34 indeed supports tubular endosome recycling. Here, we conditionally ablated Vps34 in PTCs by Wnt4- or Pax8-Cre-dependent excision of Vps34 exon 21 leading to in frame deletion (hereafter abbreviated as Vps34^Δ/Δ^). Wnt4-driven excision caused high perinatal mortality, but provided first structural clues. Pax8-driven excision did not alter mouse growth until postnatal day 14 (P14), allowing structural and functional studies. We found that Vps34^Δ/Δ^ did not prevent specification of PTC membrane polarity, but impaired apical membrane protein trafficking, thus causing a renal Fanconi-like syndrome with polyuria and low-molecular weight proteinuria, detected at P7. Vps34^Δ/Δ^ also impaired lysosome size/positioning from P7 onward, and blocked autophagy, thereby causing cell vacuolization at P14 and leading to kidney failure and death. We conclude that Vps34 is a crucial component of the trafficking machinery necessary for differentiated PTC function and is essential for overall PTC homeostasis.

## Results

### Conditional inactivation of Vps34 in PTCs

To inactivate Vps34 in kidney PTCs *in vivo*, we used a mouse line (*Vps34*^*fl*^) where exon 21, encoding a critical stretch (Ala^730^ to Thr^754^) of the Vps34 kinase domain, is flanked by *lox*P sites, allowing in-frame deletion by Cre-recombinase^45^. However, in the megakaryocyte lineage, this minimal truncation strongly decreases stability of the Vps34 protein and its associated regulatory protein Vps15^45^. For conditional deletion in kidney PTCs, *Vps34*^*fl*^ mice were crossed with Wnt4-Cre mice (mouse model A, Wnt4-Cre;*Vps34*^*fl/fl*^, further referred to as Wnt4-Vps34^Δ/Δ^) or Pax8-Cre mice (mouse model B, Pax8-Cre;*Vps34*^*fl/fl*^, further referred to as Pax8-Vps34^Δ/Δ^). Genomic excision was verified by a two-fold decrease of the amplicon spanning exon 21–22 boundary, but normal abundance of 5′ and 3′ amplicons in Pax8-Vps34^Δ/Δ^ as compared with wild-type (WT) total kidney extracts at P7 (see Supplementary Fig. S1), consistent with tissue-restricted and extensive but incomplete recombination in Pax8^+^ cells. Mosaicism provided informative pattern comparison: tubular sections of Vps34^Δ/Δ^ mice with some cells showing undistinguishable features as in WT mice were denoted by # in the figures. Evaluation of Vps34 protein expression by western blotting was not possible because two commercially available antibodies did not generate convincing Vps34 band at the expected position (100 kDa).

### Redistribution of apical membrane markers and perinatal mortality in Wnt4-Vps34^Δ/Δ^ mice

**Figure 1:**
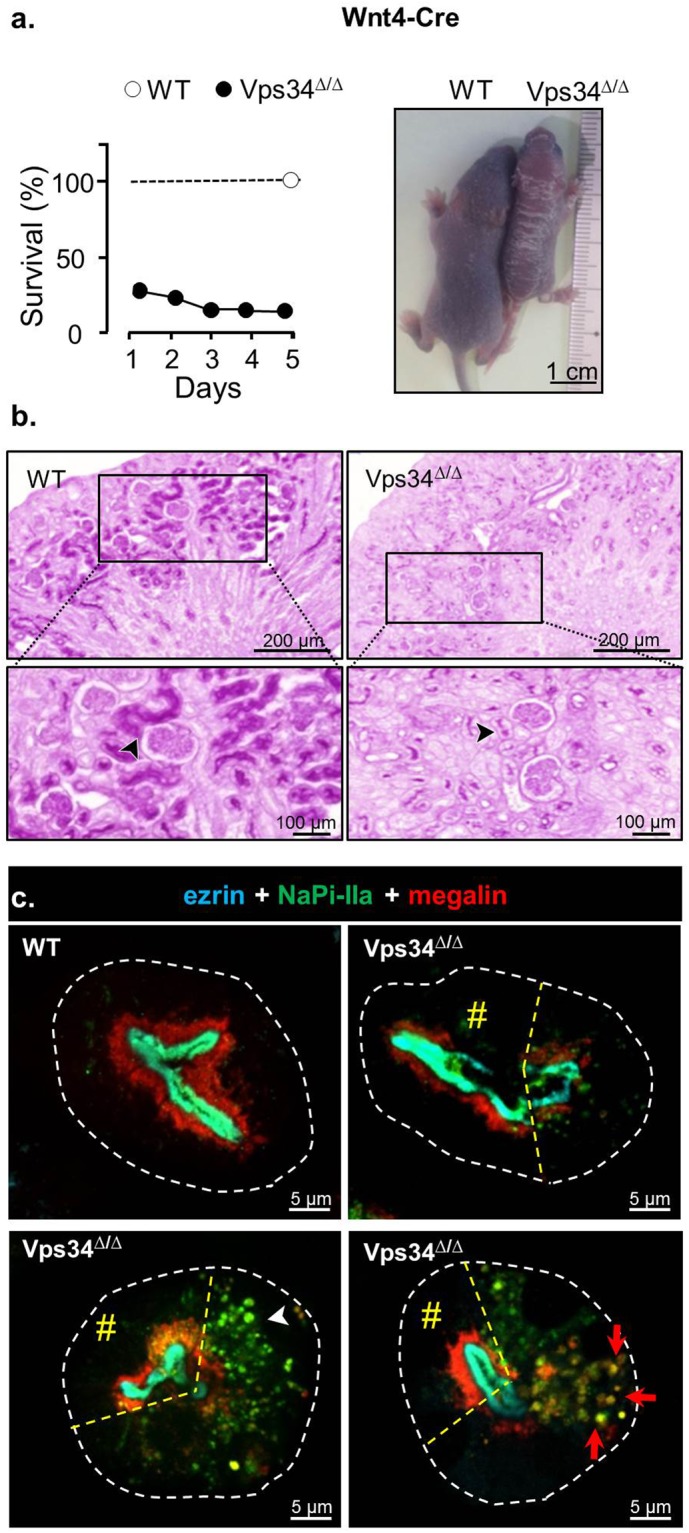
Mouse model A. Wnt4-driven excision of Vps34 exon 21 (Wnt4-Vps34^Δ/Δ^) causes perinatal kidney lesions and death. (**a**) Survival. Offspring of Vps34^fl/fl^ x Wnt4-Cre; Vps34^fl/+^ was genotyped within 24 h after birth and surviving mice were sacrificed at P5. At the left, the survival of Vps34^Δ/Δ^ mice is expressed as percentage of WT littermates (n = 25 WT pups *versus* 5 Vps34^Δ/Δ^ at P1 and 3 Vps34^Δ/Δ^ at P5, euthanized at this age). At the right, a representative surviving Vps34^Δ/Δ^ pup is compared with a WT littermate at P5. (**b**) Histology after PAS staining at P5. Boxed areas are enlarged below. Arrowheads point to apical tubular PAS staining. Note that Wnt4-driven excision alters kidney growth but is compatible with normal morphogenesis of glomeruli and kidney tubules, yet with much reduced apical PAS staining. (**c**) Confocal immunofluorescence at P5. WT (upper left) *vs* Vps34^Δ/Δ^ kidney deep (mature) cortices were immunolabeled for brush border (ezrin, cyan), NaPi-IIa (green) and megalin (red). Contours of proximal tubules recorded by bright-field microscopy are delineated by dashed white lines. Three representative views from discrete (upper right), moderate (lower left) to extensive (lower right) mis-localization of (sub)apical membrane proteins in deep cytoplasm of some PTCs contrasting with preserved localization in adjacent cells of the same tubular section shown at left of dashed dashed lines (#, mosaicism). Notice stratification in mature WT PTCs between pale green layer (full co-localization of ezrin and NaPi-IIa at brush border), and subapical red layer showing major megalin pool, with “hairy” deeper extensions. In altered Vps34^Δ/Δ^ PTCs, preferential mislocalization of NaPi-IIa yields green dots (lower left, arrowhead); shared mis-localization of NaPi-IIa and megalin generates yellow dots (lower right, red arrows). Whole cortical views are shown in Supplementary Fig. 2.

We first used Wnt4-Cre recombinase which yields virtually complete excision of the megalin-floxed gene in PTCs^46^. Genotyping at P1 indicated high perinatal mortality within the expected 25% of Vps34^Δ/Δ^ offspring from the Vps34^fl/fl^ x Wnt4-Cre;Vps34^fl/+^ intercrosses (Fig. 1a, left panel). Vps34^Δ/Δ^ survivors (5.8%) showed impaired growth and vigor, and were euthanized for study at P5. A representative survivor is shown in Fig. 1a, right panel. Vps34^Δ/Δ^ kidneys of surviving mice showed normal colour and surface but were distinctly smaller and hypotrophic. Histologically normal glomeruli and tubules were recognized, yet with weak brush border (BB) staining by PAS, suggesting impaired apical differentiation (Fig. 1b). We next immunolocalized ezrin (highly concentrated at brush border), NaPi-IIa (main Na^+^/phosphate co-transporter) and megalin (paradigm for apical receptor-mediated endocytosis^6^ and fast apical recycling pathway^8^) (Fig. 1c). In WT P5, all PTCs showed strict apical stratification between two layers: (i) ezrin and NaPi-IIa co-localized at the brush border, and (ii) megalin signal concentrated in a subapical endosomal layer, as previously reported^47–49^. As shown in Supplementary Fig. S6a, NaPi-IIa was restricted to BB in inner cortex PTCs, but also occurred as longitudinal cytoplasmic stripes in PTCs from superficial cortex, indicating an immature pattern. In Vps34^Δ/Δ^ survivors at P5, most PT sections showed coexistence of normally differentiated cells next to cells exhibiting punctate relocalization of NaPi-IIa alone, or combined with megalin throughout the cytoplasm.

We concluded that Wnt4-Cre driven Vps34 excision caused major perinatal lethality; and abnormalities in kidney cortex development and PTC apical differentiation in the surviving mice. To better analyze PTC apical differentiation and reabsorptive function in representative Vps34^Δ/Δ^ pups, we next analyzed mice with Pax8-Cre-driven Vps34 excision^42^.

### Redistribution of apical membrane markers and postnatal mortality in Pax8-Vps34^Δ/Δ^ mice

**Figure 2:**
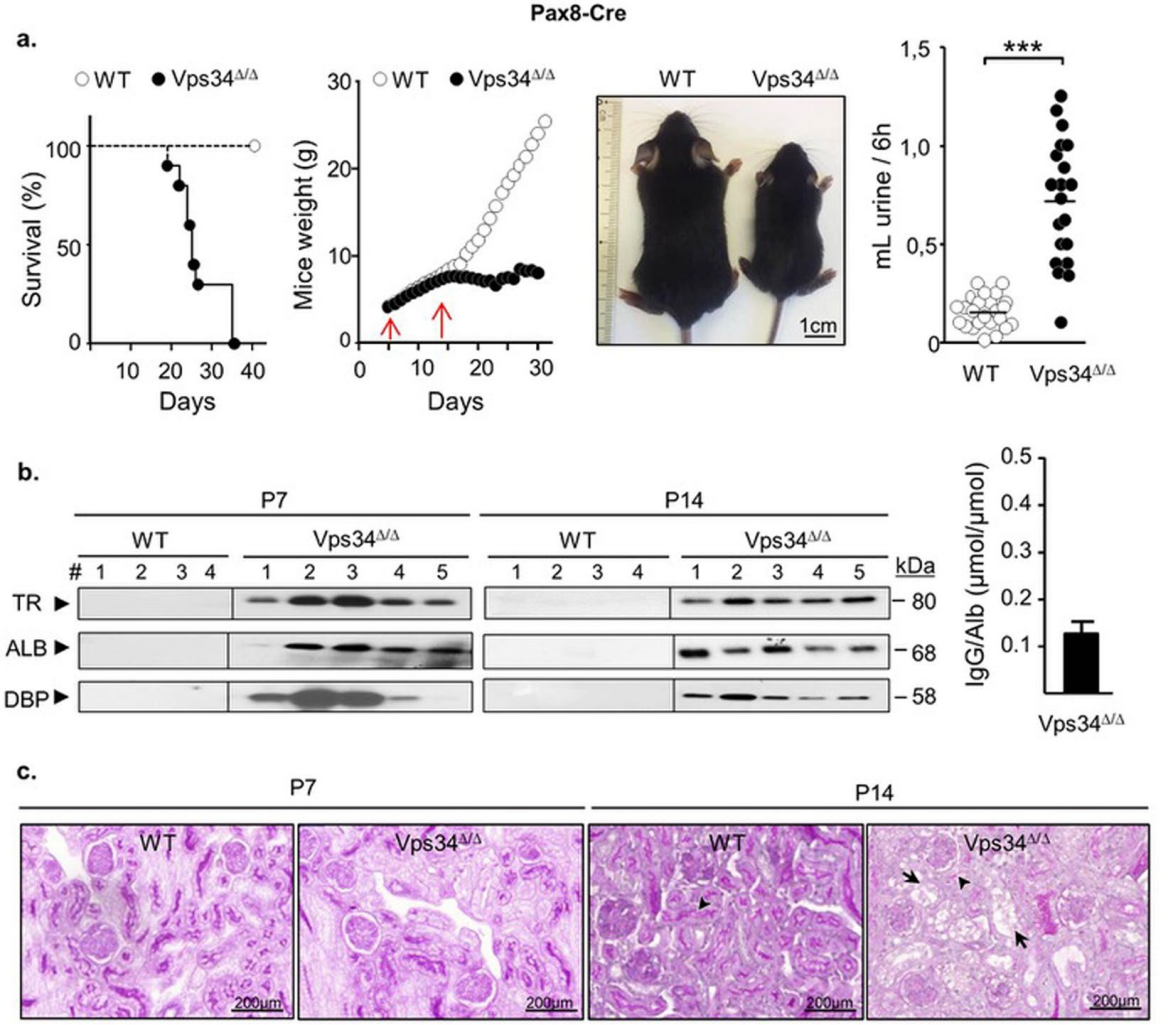
Mouse model B. Pax8-driven excision of Vps34 exon 21 (Pax8-Vps 34^Δ/Δ^) causes delayed postnatal kidney lesions and death. (**a**) Survival, growth and phenotype. In this mouse model, Vps34^Δ/Δ^ pups are born at the expected Mendelian frequency and gain weight normally until ~P14 (weaning), then level off until death. Picture shows a typical appearance at P21. Vps34^Δ/Δ^ pups are distinctly smaller but appear otherwise normal. They exhibit marked polyuria. (**b**) Western blotting of urine (n = 4 WT; 5 Vps34^Δ/Δ^), collected by bladder punction at sacrifice (P7) or upon 6-h placement in adapted metabolic cage (P14), for transferrin (TR), albumin (ALB) and vitamin D-binding protein (DBP). Loading was normalized for equal creatinine content. Positions of corresponding protein signals (in kDa) are indicated at the right. Low molecular-weight proteinuria is found in Vps34^Δ/Δ^ samples. At the right, intensity of albuminuria at P14 was compared to urinary loss of IgG, both quantified by reference to serial dilutions of normal mice plasma, and IgG/albumin molar ratio was calculated as index of selective proteinuria. (**c**) Histology after PAS staining. Strong apical PAS stain is observed in all WT PTCs (arrowhead), contrasting with paler Vps34^Δ/Δ^ pups PTCs at P7 and marked vacuolization at P14 (arrows).

Pax8-Cre is expressed two days later (at embryonic day (e) 16.5) than Wnt4-Cre activity^50^, in nephrogenic tubules and in nonvascular components of glomeruli^51^. As shown in Fig. 2a, Pax8-Vps34 mice were born at the expected Mendelian frequency (25/98) and grew normally up to ~2 weeks of age. Then, despite normal overall appearance and behavior, body weight levelled off and all pups died at ~3–5 weeks of age. We thus analyzed Vps34^Δ/Δ^ pups at P7 days (normal growth) and P14 (growth arrest; compatible for 6-h urine collections).

At P14, Vps34^Δ/Δ^ pups were markedly polyuric (~40% of body weight/day). Urinalysis (Table 1) indicated loss of glucose but preserved renal filtration at P7, then evolution to kidney failure at P14. To evaluate receptor-mediated endocytosis, urinary samples were analyzed by western blotting for the so-called low-molecular weight (LMW) proteins (transferrin, albumin, vitamin D-binding protein), characteristic of tubular proteinuria. As shown in Fig. 2b, LMW proteinuria was detected in 4/5 Vps34^Δ/Δ^ mice at P7 and in 5/5 at P14. Selectivity of LMW proteinuria by the glomerular filter was confirmed by a low (0.15) IgG/albumin molar ratio. Taken together, polyuria, glucosuria and LMW proteinuria indicated that Pax8-driven Cre deletion of Vps34 caused an early global PTC dysfunction, i.e. renal Fanconi-like syndrome.

**Table 1:** .

Weight				Urine								Plasma		
		body (g)	kidneys (mg)		volume (mL/24 h)	creatinine (mg/dL)	creatininuria (mg/24 h)	glucose (mg/dL)	glucosuria (mg/24 h)	phosphate (mg/dL)	phosphaturia (mg/24 h)		creatinine (mg/dL)	BUN (mg/dL)
**P7** ^**1**^														
WT	*n* *=* *13*	4.39 ± 0.61	29 ± 5	*n* = 4*	N/A	9.5 ± 1.5	N/A	9.6 ± 0.8	N/A	301 ± 28	N/A	*n* = *5*	0.12 ± 0.05	31.5 ± 4.5
Vps34^∆/∆^	*n* = *13*	4.12 ± 0.39	31 ± 4	*n* = 4*	N/A	8.6 ± 1.4	N/A	17.9 ± 4.9	N/A	209 ± 58	N/A	*n* = *8*	0.17 ± 0.05	53.2 ± 8.4
								P = 0.0286						P = 0.0016
**P14** ^**2**^														
WT	*n* = *6*	6.38 ± 1.05	51 ± 10	*n* = *6*	0.44 ± 0.13	11.9 ± 7.5	0.04 ± 0.03	9.7 ± 4.2	0.04 ± 0.02	386 ± 181	1.8 ± 1.1	*n* = *10*	0.11 ± 0.03	48.0 ± 14.0
Vps34^∆/∆^	*n* = *11*	5.50 ± 0.89	63 ± 13	*n* = *11*	2.06 ± 0.82	5.3 ± 2.8	0.11 ± 0.07	5.6 ± 2.2	0.09 ± 0.04	216 ± 163	3.8 ± 2.8	*n* = *10*	0.32 ± 0.18	156.8 ± 86.6
					P = 0.0011		P = 0.0119		P = 0.0256				P = 0.0019	P < 0.0001

*n = 4 pools, each of 4 pups.

Pax8-Vps34^Δ/Δ^ kidneys at P7 appeared normal and showed preserved histology, except for lack of PAS staining in ~half tubular sections (Fig. 2c). At P14, kidneys had become pale and bulging, indicating tissue swelling with reduced perfusion and were heavier (Table 1). RT-qPCR analysis displayed no changes in podocin mRNA up to P14, indicating preservation of glomeruli, but a significant decrease after P7 for AQP-1, megalin, cubilin, NaPi-IIa and SGLT-2 (indicating selective impairment in PTCs, originating from metanephric mesenchyme) and AQP-2 (in collecting ducts, originating from ureteric bud) (Supplementary Fig. S2). By histology, contrasting with preserved glomeruli, many tubular Pax8-Vps34^Δ/Δ^ sections showed loss of apical PAS staining and strong vacuolization at P14. By electron microscopy, (Supplementary Fig. S3), Vps34^Δ/Δ^ PTCs exhibited at P7 disconnected apical endosomes indicating loss of dense apical tubules. At P14, Vps34^Δ/Δ^ PTCs further showed enlarged multivesicular late endosomes/lysosomes and gigantic lysosomes/residual bodies filled with structured (undigested) material, but always devoid of mitochondrial remnants. Mitochondrial sections were preserved except in disrupted tubules. To look at tight junction integrity, we performed tracing from the interstitium of ruthenium-red, a readily diffusing electron-dense molecule providing labelling of accessible membranes (Supplementary Fig. S4). Ruthenium-red labeled the entire basolateral membrane of Vps34^Δ/Δ^ PTCs and was arrested at the position of tight junctions, even at strongly vacuolated cells, indicating a tight/sealed epithelium.

**Figure 3:**
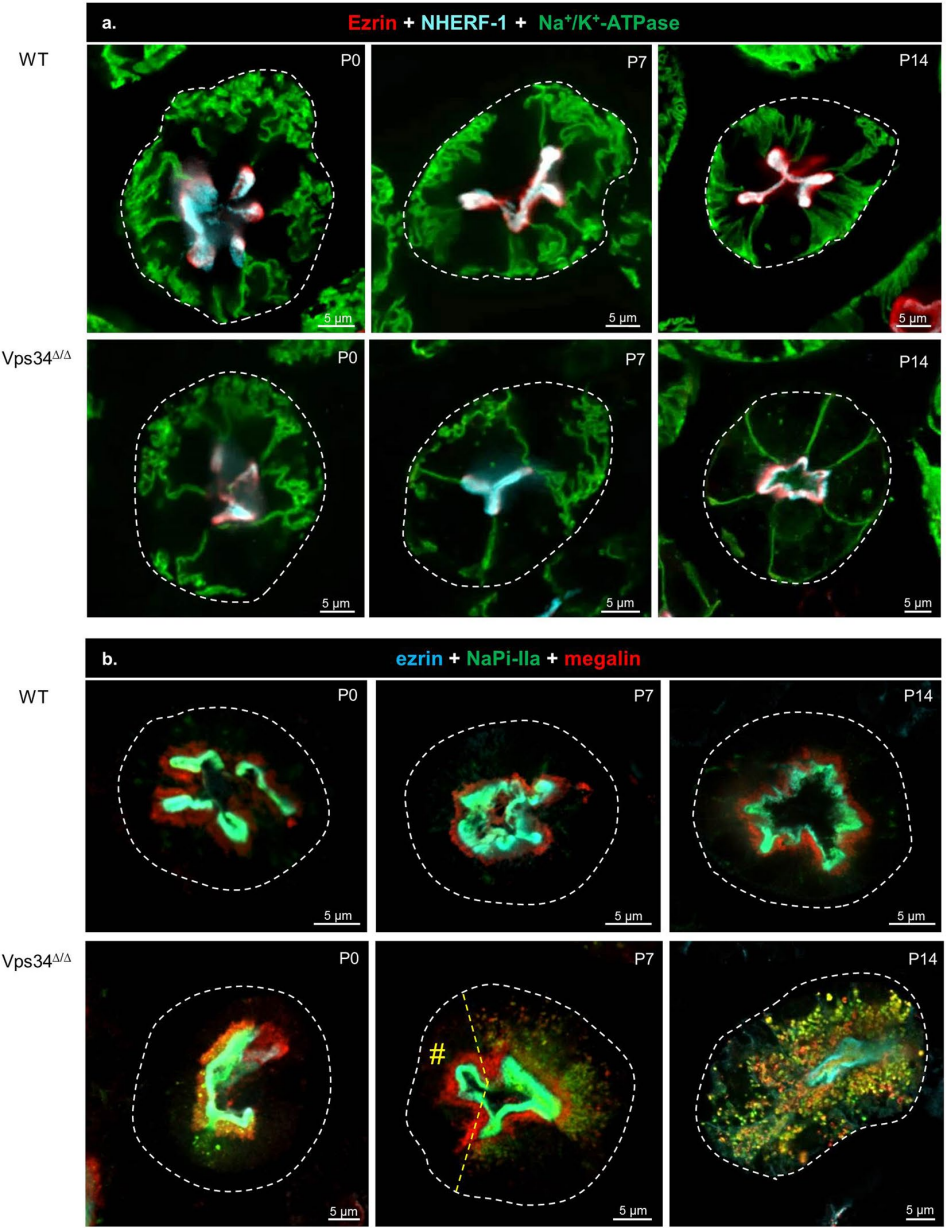
Pax8-Vps34^Δ/Δ^ mice show preserved apico-basal polarity in kidney proximal tubules but increasing mislocalization of megalin and NaPi-II from P7 to P14. (**a**) Confocal imaging of epithelial polarity markers: ezrin (apical brush border; red), NHERF-1 (apical PDZ scaffold; cyan), Na^+^/K^+^-ATPase subunit α1 (basolateral domain; green). Almost perfect colocalization of ezrin with NHERF-1 generates an apical white layer at P7 and P14, irrespective of conditional Vps34 excision. In WT PTCs and in most Vps34^Δ/Δ^ PTCs up to P7, notice extensive invaginations of basal membrane labeled for Na^+^/K^+^-ATPase. These structures have vanished in Vps34^Δ/Δ^ PTCs at P14. (**b**) Confocal imaging of apical transmembrane proteins: megalin (red), NaPi-IIa (green), by reference to ezrin (cyan). In WT PTCs, NaPi-IIa perfectly colocalizes with ezrin (pale green signal) while megalin is concentrated in a subapical layer. In Vps34^Δ/Δ^ PTCs, megalin and NaPi-IIa are progressively mislocalized to separate (red, green) or common (yellow) deep cytoplasmic dots. Yellow dashed line indicates mosaicism with preserved cell indicated by #. For cubilin and SGLT-2, see Supplementary Fig. 5.

We next examined apical-basal PTC polarity by confocal immunofluorescence for the brush border linker, ezrin, and the apical domain marker, NHERF-1 (PDZ-scaffold for transporters), combined with the basolateral membrane marker, Na^+^/K^+^-ATPase, and its scaffold, ankyrin-G (Fig. 3a). Both WT and Vps34^Δ/Δ^ pups showed extensive apical co-localization of NHERF-1 with ezrin and perfect segregation of these proteins from basolateral domains. In WT pups, basal infoldings bearing Na^+^/K^+^-ATPase were tortuous at P0 and P7, but orderly perpendicular (comb-like) at P14, suggesting terminal basal differentiation. Basal infoldings were less conspicuous in Vps34^Δ/Δ^ compared to WT mice at P7 and disappeared at P14, at which time Na^+^/K^+^-ATPase was mostly at lateral membranes with a straight radial orientation, consistent with cell swelling. We concluded that Vps34 deletion in PTCs preserved apico-basal polarity, but altered terminal basolateral differentiation.

In the context of renal Fanconi-like syndrome, we next assessed by confocal multiplex immunofluorescence NaPi-IIa (non-recycling, green) and megalin (recycling, red) by reference to ezrin (brush border, cyan) (Fig. 3b). In WT pups up to P14, we confirmed the extensive co-localization of NaPi-IIa with ezrin, thus at brush border, and the distinct subapical megalin layer. In contrast, in Vps34^Δ/Δ^ cells, NaPi-IIa and megalin appeared well apically localized in differentiated PTCs at P0 and became mislocalized into cytoplasmic compartments at P7 and more severely at P14. Again, mosaicism provided informative intercellular comparisons. Mislocalization was first detected for NaPi-IIa (mostly green punctae), then extended to megalin (mostly yellow punctae). As shown in Supplementary Fig. S5, a similar mislocalization was observed for the main PTC glucose transporter, SGLT-2^3^, and the tandem endocytic receptor, cubilin^52^. Considering the marked heterogeneity of protein relocation, we performed morphometry based on NaPi-IIa, which showed the earliest mislocalization and allowed to distinguish immature (presence of lateral cytoplasmic stripes) from mature pattern (brush border only), (Supplementary Fig. S6b). As shown in Supplementary Fig. S7, tubulogenesis was not arrested in Vps34^Δ/Δ^ kidney (comparable proportion of immature over mature patterns), and mislocalization of NaPi-IIa at P14 proved highly significant. We concluded that Pax8-Cre-mediated Vps34 deletion was compatible with tubulogenesis and preserved overall PTC polarity but caused extensive mislocalization of both recycling and non-recycling apical membrane proteins into cytoplasmic compartments.

### Apical endocytosis is severely impaired in Pax8-Vps34^Δ/Δ^ PTCs

**Figure 4:**
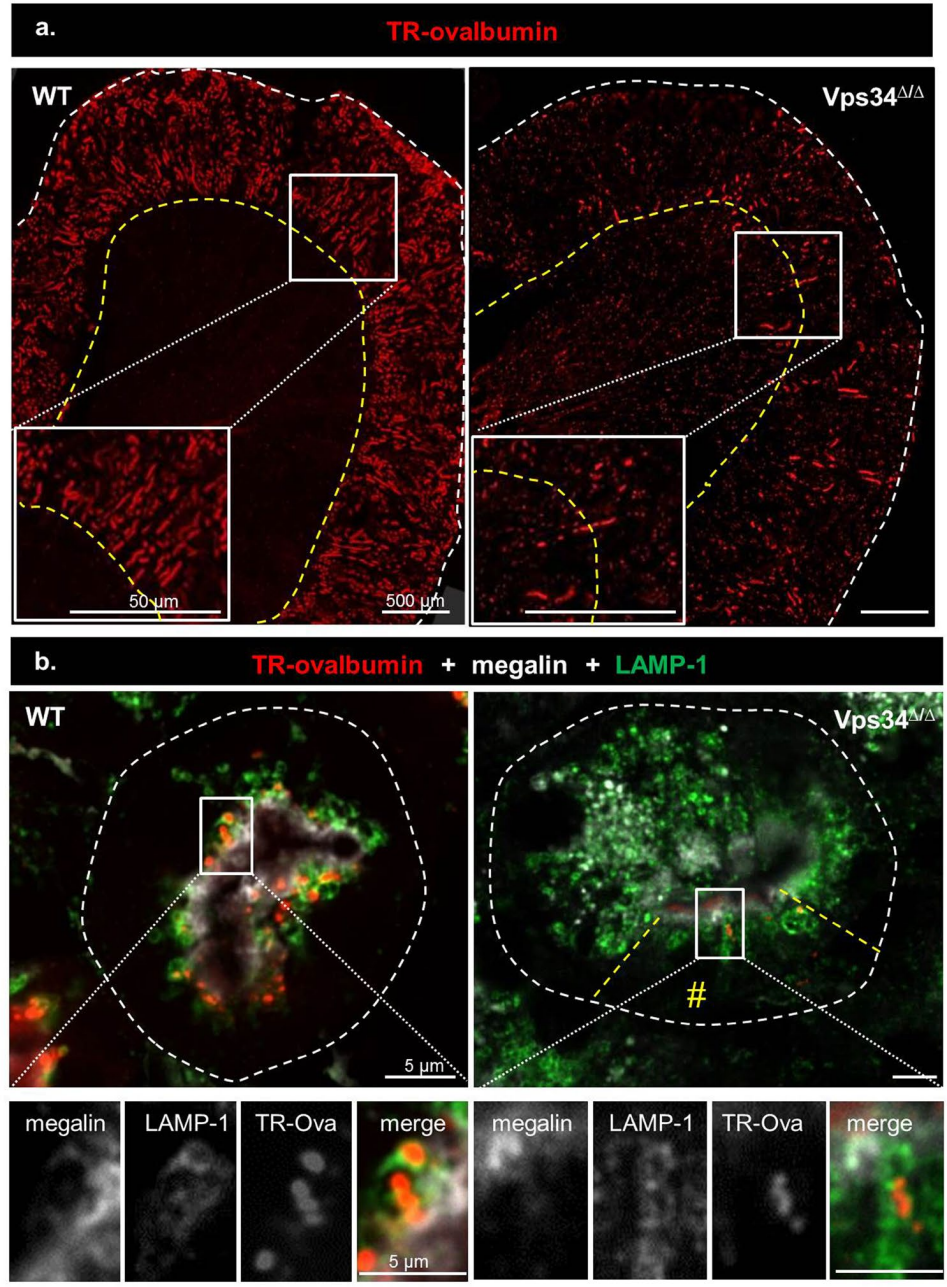
Vps34 deletion impairs acute endocytosis of injected ovalbumin-texasRed. WT or Vps34^∆/∆^ mice were injected at P14 with 180 µg TexasRed-labelled ovalbumin (TR-Ova) in the orbital plexus and euthanized at 20 min. Kidneys were fixed by immersion and whole cryostat sagittal sections were collected. (**a**) Low-power view of TR-Ova signal by stitched images of the two upper thirds of the sagittal sections recorded by Zeiss Mirax Midi and examined for red emission. Dashed white and yellow lines define the kidney contours and the cortico-medullary junctions, respectively. Boxes are enlarged below. Notice in WT mice the homogenous cortical signal indicating extensive recapture by all PTCs, with abrupt arrest at the cortico-medullary boundary (between cortex and outer stripe of outer medulla, OSOM). In Vps34^∆/∆^, much fewer PTCs are labeled and red signal extends deeper into the OSOM. (**b**) High magnification analysis by confocal microscopy with triple PTC imaging for TR-ovalbumin as endocytic cargo (red), megalin as endocytic receptor (white) and LAMP-1 as membrane marker of lysosomes where undigested tracer accumulates and free Texas Red is trapped (green). PTCs are circumscribed by broken white lines. Fields in the boxed rectangles are shown enlarged below with separate emission channels in white then merged channels in colors. Preserved PTC due to mosaicism in cKO is indicated by two radial broken yellow lines. In WT PTCs, megalin signal is restricted to a continuous apical layer. Intensely red dots indicating endocytosed TR-ovalbumin are mostly surrounded by LAMP-1, still in the subapical layer, indicating extensive endocytic uptake and transfer to lysosomes. In Vps34^∆/∆^ PTCs, LAMP-1 objects are much more numerous and become mislocalized to the deeper cytoplasm. Megalin has mostly disappeared from the apical layer and is instead redistributed into cytoplasmic dots colocalizing with LAMP-1. TR-Ova is not detected, except in the few preserved PTCs at the bottom of the section, where megalin and LAMP-1 show normal localization and intensity.

To directly bridge LMW proteinuria with mislocalization of endocytic receptors away from the apical membrane, we looked at endocytic cargo uptake. First, we performed at P14 a 20-min *pulse* study of Texas-Red-labeled ovalbumin (TR-ova), validated to trace PTC endocytosis and lysosomal accessibility in PTCs; and its alteration in disease^49^. As shown by low-power views of sagittal kidney sections (Fig. 4a), the entire cortex of WT mice showed intense and homogenous radial TR-ova signal, with abrupt arrest at the cortico-medullary junction. In Pax8-Vps34^Δ/Δ^ kidneys, much fewer nephrons were labeled but signal extended into the outer stripe of the outer medulla, indicating compensatory uptake by more distal cells. These sections were further immunolabeled for megalin as endocytic receptor and LAMP-1 as membrane marker for lysosomes, the final compartment of the degradative pathway where free Texas red is also sequestered, and analysed at high magnification. In WT tissue, TR-ova was concentrated in apical dots below the continuous megalin layer, largely co-localizing with LAMP-1. In Pax8-Vps34^Δ/Δ^ PTCs showing megalin mislocalization, no TR-ova signal was detected.

We also assessed by immunofluorescence the steady-state albumin content, as alternative reporter of PTC endocytic activity^48^. As shown by Supplementary Fig. S8, we found a severe reduction of albumin recapture in Vps34^Δ/Δ^ PTCs (protected adjacent PTCs without deletion being positive controls). Thus, mislocalization of endocytic receptors (and presumably solutes co-transporters) provided a straightforward explanation for the Fanconi-like syndrome.

### Disruption of apical Rab11- and perinuclear galectin-3-compartments in Pax8-Vps34^Δ/Δ^ PTCs provides two mechanistic clues for apical membrane protein redsistribution

**Figure 5:**
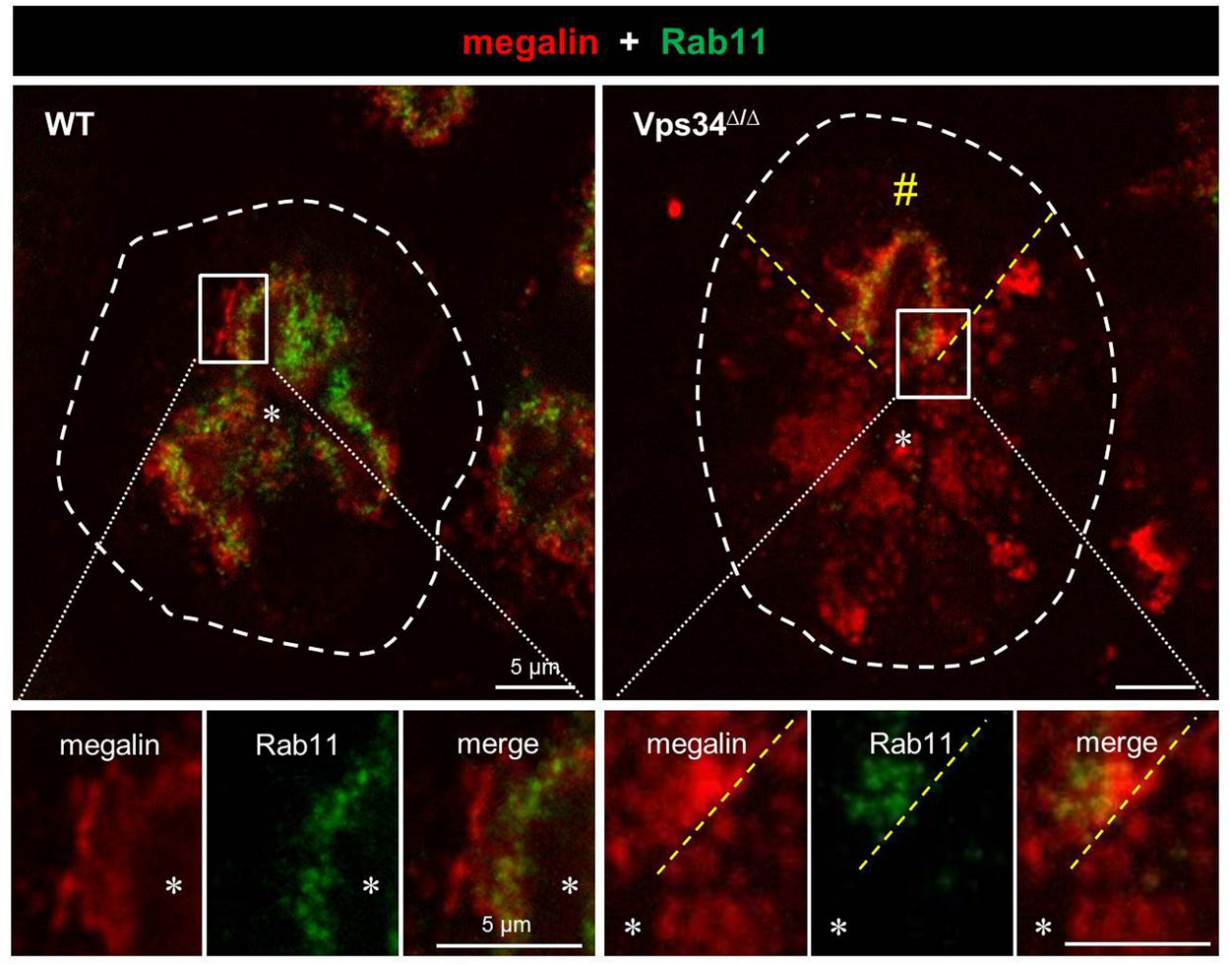
Vps34 deletion in PTCs disrupts the Rab11-labeled, apical recycling endosomal compartment. Kidney sections from WT and Vps34^∆/∆^ P14 pups were double-immunolabeled for megalin (red) and Rab11 (green). Broken white lines delineate PTC contours. The pair of radial broken yellow lines defines in the upper field unusual PTCs with normal appearance in Vps34^∆/∆^ mice, attributed to mosaicism (notice preservation of continuous megalin layer without dotty cytoplasmic redistribution, contrasting to other PTCs of the same tubule). Boxed rectangles are enlarged below. In WT PTCs, Rab11 antibodies produce a specific finely dotty apical signal in the most apical half of the continuous megalin layer (Lu, PTC lumen), labelling the apical, rapidly recycling endosomal compartment. In Vps34^∆/∆^ PTCs showing megalin mislocalization into deep cytoplasmic dots (lower cells, best visible below the yellow broken line in the enlargements), the Rab11 signal is not detectable, indicating disruption of the apical recycling endosomal compartment.

In OK cell lines as PTC model, acute Vps34 inhibition reversibly abrogates apical megalin recycling^44^. Since the fast apical megalin recycling pathway critically depends on Rab11^8^, we thus examined by immunofluorescence whether Vps34 deletion would impact the Rab11 apical recycling compartement. As shown in Fig. 5, Rab11 occurred in WT PTCs as a dense punctate apical compartment, partially overlapping with the megalin signal and extending closer to the lumen, as expected for rapidly recycling apical endosomes/dense apical tubules. Vps34 excision totally disrupted this compartment. The sharp mosaique contrast between affected and preserved PTCs evidenced the cell-autonomous effect of Vps34 deletion. Disruption of the Rab11 apical compartment thus provided a straightforward explanation for suppressed megalin and cubilin recycling.

However, this could not explain mislocalization of the non-recycling NaPi-IIa and SGLT-2, anchored to the NHERF-1/apical actin scaffold. We therefore looked for a primary defect of apical biosynthetic trafficking. Non-raft dependent apical sorting of N-glycan bearing glycoproteins at the trans-Golgi depends on luminal clustering by galectin-3^10^. By immunofluorescence, galectin-3 in WT PTCs showed a remarkable perinuclear ribbon-like pattern, suggesting its concentration by retrograde trafficking into the Golgi/TGN complex. This pattern was reminiscent of galectin-3-GFP in Cos7 cells, especially upon TGN retention by a 20 °C block (see ref.^10^, Fig. 2b). In Vps34^Δ/Δ^ PTCs, the perinuclear galectin-3 ribbons disappeared into dispersed dots, suggesting disruption of the biosynthetic pathway supporting apical trafficking of N-glycan bearing membrane proteins, including NaPi-IIa and SGLT-2.

### In Pax8-Vps34^Δ/Δ ^PTCs, late endosomes/lysosomes are mislocalized to basal perinuclear cytoplasm

**Figure 6:**
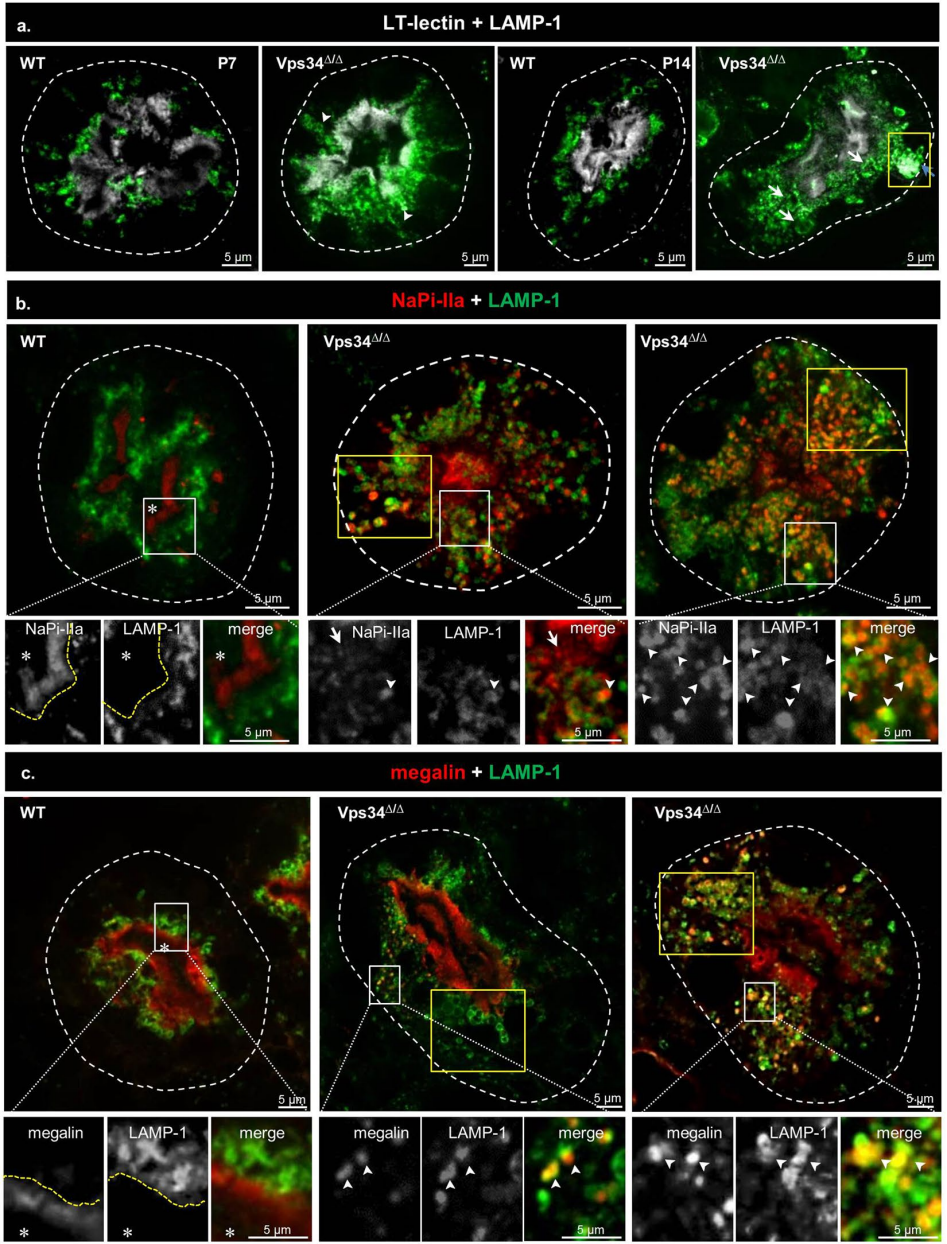
Mislocalization of late endosomes/lysosomes, followed by vacuolisation, and progressive redistribution of NaPi-IIa and megalin to late endosomes/lysosomes in Pax8-Vps34^Δ/Δ^. (**a**) Confocal imaging of LAMP-1 (green) by reference to PTC apex (lotus-tetragonolobus lectin, LT-lectin, white) and basal profile (dotted white contours). Arrowheads, radial LAMP-1 redistribution; arrows, late endosomes/lysosomes vacuolization; yellow box, huge lysosome filled with LAMP-1 (see also Fig. 7b,c). (**b**,**c**) NaPi-IIa (**b**, red) and megalin (**c**, red) are compared to LAMP-1 (green). Areas boxed in white are enlarged below with split or merge channels. Areas boxed in yellow for Vps34^Δ/Δ^ mice illustrate clusters of basally-located late endosomes/lysosomes. Yellow dotted lines in inserts distinguish two concentric apical cytoplasmic layers in PTCs. Asterisks, tubular lumen. In WT PTCs, the diffuse NaPi-IIa layer (brush border) is separated by unstained zone from deeper LAMP-1 zone made of discrete small dots. The diffuse megalin layer contacts, yet does not overlaps with, the LAMP-1 layer. In Vps34^Δ/Δ^, NaPi-IIa redistributes to deep cytoplasmic dots, many also labeled for LAMP-1 (arrowheads, yellow signal). Megalin may retain its normal subapical location even in cells where LAMP-1-labeled late endosomes/lysosomes extend into the basal cytoplasm, or is mislocalized to mispositioned lysosomes like NaPi-IIa.

**Figure 7:**
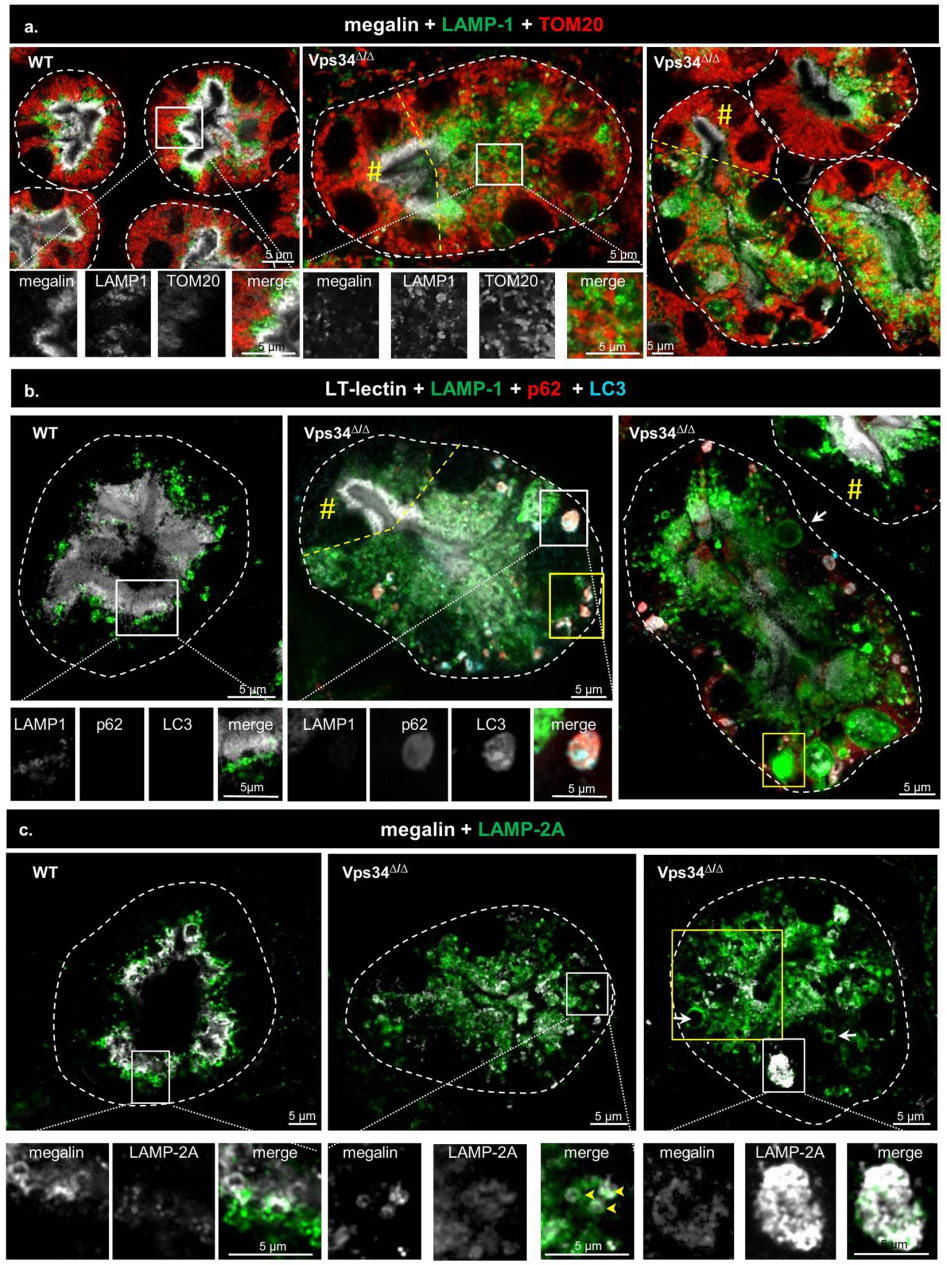
Abrogation of macro-autophagy in Pax8-Vps34^Δ/Δ^ PTCs. (**a**) Mitophagy. In WT PTCs, megalin (white), late endosomes/lysosomes (LAMP-1, green) and mitochondria (TOM20, red) define distinct layers. In Vps34^Δ/Δ^ PTCs, note the loss of stratification (central panel, right to dashed yellow line; right panel, below dashed yellow line). TOM20 is never detected in mislocalized late endosomes/lysosomes. (**b**) Selective p62-dependent autophagy. In WT PTCs, LAMP-1-labeled late endosomes/lysosomes (green) in subapical zone are segregated from the LT-lectin-labeled apical cytoplasmic layer (white). No p62 (red) and LC3 (blue) puncta is visible. Vps34^Δ/Δ^ PTCs sections usually show several punctae double-labeled for p62 and LC3 in the basal cytoplasm (compare with preserved cell indicated by yellow #). For P7, see Supplementary Fig. 7. (**c**) Chaperone-mediated autophagy. In WT PTCs, LAMP-2A labels similar structures as LAMP-1, polarized in the subapical cytoplasm, and likewise segregated from megalin. In Vps34^Δ/Δ^, LAMP-2A-surrounded structures are much more abundant, extend into basal cytoplasm where they are often enlarged (arrows in right panel; area boxed in yellow) and become labeled for megalin (arrowheads in central panel). The enlarged boxed area shows lysosomal lumen filled by LAMP-2A-labeled objects.

To define the fate of apical membrane proteins once mislocalized into the cytoplasm, we first looked at lysosomal position, since Vps34 suppression or inhibition in cell culture was reported to cause late endosome/lysosome perinuclear mispositioning^53^. As shown in Fig. 6a, LAMP-1 immunolabelling in WT PTCs was restricted to a distinct subapical zone, below the megalin-enriched layer. Vps34 deletion strongly increased LAMP-1 signal (see also Fig. 7b), as reported in Vps34 KO podocytes^40,41^. In Pax8-Vps34^Δ/Δ^ PTCs, LAMP-1 organelles became basally located and enlarged (P14, arrows). These data are consistent with multivesicular body swelling and the huge residual bodies evidenced by electron microscopy (Supplementary Fig. S3). This observation is also consistent with the reported enlargement of LAMP-1-labeled late endosomes/lysosomes in Vps34 KO MEFs^37^ and upon pharmacological inhibition of Vps34 by SAR405 in cultured cells^54^. Occasionally, LAMP-1 labelling filled the lumen of huge basal structures (boxed in Fig. 6a; see also below, small boxes in Fig. 7b,c), corresponding to large residual bodies as identified by electron microscopy and suggesting defective LAMP-1 catabolism within lysosomes. A good marker of endolysosomal damage is provided by galectin-3 redistribution into their lumen^11^. However, we never detected galectin-3 co-localization with LAMP-1 in Pax8-Vps34^Δ/Δ^ PTCs, indicating endolysosome membrane integrity.

### In Pax8-Vps34^Δ/Δ ^PTCs, apical markers become redistributed to mispositioned lysosomes

We next performed double-labelling studies of LAMP-1 with NaPi-IIa (Fig. 6b) or megalin (Fig. 6c). In WT mice, NaPi-IIa (brush border) and megalin (predominating pool below brush border) were fully resolved from the subapical LAMP-1 zone, defining a triple stratification (see inserts). In Pax8-Vps34^Δ/Δ^ PTCs, redistributed NaPi-IIa and megalin became partially detected in LAMP-1-labeled, basally mispositioned late endosomes/lysosomes. Together, these data indicate that, as a result of defective Vps34-dependent apical targeting, transporters and endocytic receptors were mislocalized to lysosomes in Vps34^Δ/Δ^ mice where they accumulated probably due to a defective proteolysis, as previously suggested^54^.

### Macroautophagy is abortive in Pax8-Vps34^Δ/Δ^ PTCs

We finally speculated that mislocation of apical transporters, presumably including amino-acid transporters, combined with suppression of macroautophagy, should cause a severe stress and trigger compensatory mechanisms. We hypothesized that without such adaptation, cellular homeostasis would be impaired, causing cell swelling and death. In view of alternative mechanisms for PI3P production^21–23,28^, and for entry into macroautophagy^55^, we first determined whether macroautophagy was altered in Vps34^Δ/Δ^ PTCs by looking for co-localization at LAMP-1 organelles of the outer mitochondrial membrane protein, TOM20, as validated index of mitophagy^56^. As shown in Fig. 7a, TOM20 labeled elongated mitochondria in the basal cytoplasm of WT PTCs. In Vps34^Δ/Δ^ PTCs at P14, TOM20-labeled structures appeared smaller and never showed colocalization with LAMP-1 organelles, and/or containing megalin (insert of central panel in Fig. 7a), compatible with full arrest of the mitophagic flux.

We also assessed p62-mediated autophagy of aggregated polyubiquitinylated cytosolic proteins^31^. To this aim, we performed quadruple fluorescent labeling using p62, LC3 as autophagic marker, LAMP-1 and *Lotus Tetragonolobus* lectin as signature of PTCs and general marker of apical membrane structures (brush border and apical endosomes). As shown in Fig. 7b, p62 and LC3 punctae were essentially absent from WT PTCs. In contrast, small punctae, all double-labeled for p62 and LC3, could be detected scattered in the cytoplasm of Vps34^Δ/Δ^ PTCs at P7 (for time-dependent progression of alterations, see Supplementary Fig. S10). At P14, much larger punctae, mostly double-labeled for p62 and LC3, were easily found in the basal cytoplasm of Vps34^Δ/Δ^ PTCs at P14, but again were never detected in LAMP-1-labeled structures. We concluded that LC3 could be recruited on p62 aggregates but that progression to autophagosome maturation and lysosomal fusion was arrested. This suggested that deletion of Vps34 in PTCs abrogated the autophagic flux, thus suppressing amino acid supply derived from autophagy completion.

Since chaperone-mediated autophagy (CMA) (i) can be up-regulated when macroautophagy is impaired^33^; (ii) relies on direct translocation of individual chaperoned proteins across the lysosomal membrane by LAMP-2A transporter; and thus (iii) does not depend of *de novo* membrane formation^32^, we assessed CMA in Vps34 mutant PTCs. As shown in Fig. 7c, LAMP-2A-labeled structures were indeed more abundant in Vps34^Δ/Δ^ PTCs at P14 compared to WT controls and LAMP-2A extensively co-localized with LAMP-1. There was no preferential expression of the CMA-specific LAMP-2A isoform over LAMP-2B isoform as measured by RT-qPCR (data not shown). Thus, we could not find evidence that Vps34^Δ/Δ^ PTCs could compensate defective macroautophagic flux by triggering CMA.

## Discussion

Here, we tested the role of the Vps34 isoform of PI3K on the differentiation and maintenance of PTCs *in vivo*, by conditional deletion using Cre driven by two different promoters expressed at different time points during embryonic development. Our results show that Vps34 deletion allows proper apico-basal specification but causes primary mislocalization of apical membrane solute carriers and endocytic receptors, resulting in an early Fanconi-like syndrome. Vps34^Δ/Δ^ PTCs also show several lysosomal alterations and abortive autophagy, with the eventual impact on PTC homeostasis leading to fatal kidney insufficiency.

Although no mosaicism was reported when inactivating Vps34^fl/fl^ alleles in podocytes with Podocin- or Nephrin-driven Cre recombinase^40,41^, mosaicism was evident in tubular cells upon Pax8-Cre driven recombination. We consider unlikely this was due to the modified Vps34 locus itself, because (i) the floxed 21 exon used here was in the same region as in previously reported Vps34 KO mice^40,41^, and (ii) we observed similar mosaicism with our Pax8-Cre line on the Rosa26 locus (Rosa26-LSL-EYFP, data not shown). In addition, using indirect Pax8-driven deletion of mTORC components, Grahammer and collaborators^57^ also observed evident mosaicism in kidney tubules. We therefore favor the view that, in the rapidly expanding PTC pool, Pax8 promoter elements do not yield sufficient Cre activity in every tubular cell. Since Wnt4-Cre-mediated megalin recombination is virtually complete in adult mice^46^, mosaic Wnt4-Cre recombination of Vps34 locus might also be surprising, but again could be explained by the very young age of pups analysed (P5). Selectivity of tubular excision of Pax8-Cre was confirmed by preserved podocin expression up to P14, but decreased AQP-1 expression from P7 to P14. Loss of AQP-2 at P14 might indicate secondary effects.

Besides incomplete genetic recombination, phenotypic tissue heterogeneity upon Vps34 excision could be attributed to metabolic (compensatory) mechanisms of PI3P production. Although the Vps34-lipid kinase is the main producer of PI3P, other lipid kinases (e.g. class II PI3K isoforms) are capable of catalyzing this reaction^23^ and dephosphorylation of PI3,4P_2_ or PI3,5P_2_ also lead to PI3P production^21,22,28^.

Kidney PTCs are a paradigm of highly differentiated absorptive epithelium, reflected by concentric stratification of organelle layers from the apical to the basal pole, well-documented by electron microscopy^1^. In WT PTCs, immunofluorescence showed exclusive signal for NaPi-IIa to the brush border, mixed signal for SGLT-2 at the brush border and an upper subapical layer, and prominent signal for megalin and cubilin at a comparable upper subapical layer, as previously reported in mice and rats^47–49^. These distinct subcellular patterns of transporters and endocytic receptors reflect the balance between the surface pool (not readily detected by immunofluorescence for megalin and cubilin) and the prevailing recycling pool at apical endosomes *in vivo*. In mouse PTCs, late endosomes/lysosomes are localized in a slightly deeper zone, just below the dense megalin/cubilin endosomal layer and vacuoles. We identified by immunofluorescence a major pool of PI3P at the endosomal/lysosomal interface, where Rab11-rich endosomes have also been described^58^. Rab11 endosomes are the main compartment for apical membrane sorting in polarized MDCK cells^8^.

We found that the main early effect of Vps34 inactivation in PTC was the mislocalization of NaPi-IIa, SGLT-2, megalin and cubilin into cytoplasmic organelles, directly accounting for the renal Fanconi-like syndrome. At P14, mislocalization was further compounded by decreased expression, reflecting loss of cell homeostasis. Mislocalization of surface components undergoing multiple fast cycles such as megalin can be explained by defective recycling due to observed disruption of the Rab11 compartment. In addition, disruption of the galectin-3 compartment which governs N-glycan-dependent apical biosynthetic targeting, provides a tentative explanation for NaPi-IIa and SGLT-2 mistargetting. Furthermore, since membrane biosynthetic and endocytic pathways intersect at endosomes where polarity signals are primarily decoded^14^, endosomal misorientation of newly synthesized NaPi-IIa into non-recycling apical components would provide a second explanation. Apical PTC trafficking defects related to altered endosomal acidification or inositol-5-phosphatase acting on endosomes are known to cause Fanconi syndrome in Dent and Lowe diseases^16–18,21^, but further *in vitro* and *in vivo* studies are required for comprehensive molecular understanding of apical trafficking machineries. Surprisingly, PTC apico-basal specification appeared perfectly preserved in Vps34^Δ/Δ^ mice. This contrasts with 3D epithelial organoids, where pharmacological inhibition of endosomal PI3K was recently reported to impair apical polarity, resulting into multilumen-cysts^59^.

Peripheral positioning of late endosomes/lysosomes has emerged as central feature for the regulation of cell homeostasis by mTORC1^60^. Vps34/PI3KC3 regulates this positioning by tethering late endosomes/lysosomes to the anterograde/apical directed microtubule motor, kinesin-1, via the PI3P-binding proteins, FYCO-1 and protrudin^53,61^. However, to the best of our knowledge, the relation between mislocalization of late endosomes/lysosomes, mTORC1 signalling and autophagy has not been documented in polarized epithelia. We have taken advantage of mouse PTC stratification and found that Vps34 inactivation also caused basal/perinuclear lysosomal mispositioning in a polarized epithelium *in vivo*. However, mispositioning failed to promote autophagy completion since PI3KC3 is an essential component of autophagy initiation machinery.

In addition to vesicular trafficking defects, Vps34 deletion also caused functional impairment of late endosomes/lysosomes, as reflected by swelling and lysosomal accumulation of undigested material (electron microscopy; retention of various proteins identified by immunofluorescence). We also found that Vps34 inactivation in PTCs impaired autophagic flux (absence of TOM20 colocalization with LAMP-1 indicating lack of mitoghagy completion; colocalization of p62 with LC3 but not LAMP-1, demonstrating abortive autophagy of ubiquitylated cytosolic proteins). All these observations are consistent with *in vitro* evidence that Vps34 inhibition causes LAMP-1 organelle vacuolization and impairs maturation of the lysosomal enzyme, cathepsin D^54^. As expected, impaired macroautophagy was observed in Vps34^Δ/Δ^ PTCs and alternative pathways to produce PI3P or to initiate macroautophagy^30^ were clearly insufficient to support detectable autophagy in Vps34^Δ/Δ^ PTCs. Likewise, CMA could not compensate for abortive macroautophagy. Suppression of autophagy likely explains the loss of cellular homeostasis. PTC vacuolization and swelling resulted into increased intrarenal tension, as reflected by increased kidney size and weight, organ bulging and decreased perfusion, eventually leading to kidney insufficiency and death. These secondary effects were not detected at P7 but obvious at P14, thus calling for caution on interpretation at the late stage.

## Materials and Methods

### Mice

Vps34^fl/fl^ mice and Wnt4-Cre mice have been described^45,62^. Pax8-Cre mice were obtained from Dr. M. Busslinger^51^. Vps34^fl/fl^ mice were crossed with Wnt4-Cre;Vps34^fl/+^ or Pax8-Cre;Vps34^fl/+^ mice to generate conditional targeted excision of Vps34 exon 21 in the proximal tubules of 25% of offspring. Of note, Wnt4-Cre also causes excision of floxed alleles in the pituitary gland and Pax8-Cre in thyrocytes. Vps34^fl/fl^ mice were used as control, denoted WT. Wnt4-Cre or Pax8-Cre driven Vps34^fl/fl^ were denoted Vps34^Δ/Δ^. Mice were raised and treated according to the NIH Guide for Care and Use of Laboratory Animals, and experiments were approved by the local Ethical Committee of UCL (2016/UCL/MD/005).

### Plasma, tissue collection and histology

Blood was collected at sacrifice, under anesthesia by xylazine 2% and ketamine 50 mg/ml, by heart (P7) or eye sinus puncture (P14). Kidneys were excised, decapsulated, and weighted then hemi-sagittally sectioned kidneys were fixed by immersion in neutral-buffered formaldehyde (4% F) at 4 °C under stirring overnight. Samples were paraffin-embedded or equilibrated overnight in 20% sucrose and embedded in Tissue-Tek Optimal Cutting Medium (Sakura Finetek) for 5 µm-thick cryostat sections.

### Kidney function and western blotting

P14 pups were kept in metabolic cages for a duration of 6 h (10.00 a.m. to 4.00 p.m.) under adapted access to drink and urine was collected on ice with protease inhibitors cocktail (CompleteTM Protease Inhibitor Cocktail Tablets, Roche). Urine was weighted. Urinary creatinine was measured by improved colorimetric Jaffe method (QuantiChromTM BioAssay System). Glucosuria and phosphaturia were measured by automated assays by Roche Cobas Integra 6000. Plasma levels of creatinine and urea were measured on a COBAS 6000 C501 device (Roche-Hitachi^®^). For western blotting, the equivalent of 0.5 µg creatinine urine was resolved on precasted gels (Mini protean TGX 4–15%, 456–1083, Bio Rad). Antibodies are listed in Supplementary Table II.

### Immunofluorescence

Immunofluorescence was performed on 5 µm-thick frozen sections or on 6 µm-thick paraffin sections. Antigen retrieval was promoted in citrate buffer, pH 6.0, at 98 °C for 20 min using a Lab Vision Pretreatment Module™ (Thermo Scientific). After permeabilization with PBS/0.3% Triton-X100 for 5 min, non-specific sites were blocked by 1-h incubation in PBS/0.3% Triton-X100 with 10% bovine serum albumin (BSA) and 3% milk, followed by primary antibodies in blocking buffer at 4 °C overnight. After extensive washing, sections were incubated with the indicated AlexaFluor-secondary antibodies in 10% BSA/0.3% Triton-X100 at room temperature for 1 h, extensively washed, mounted with Faramount Aqueous Mounting Medium (Dako) and imaged on a spinning disk confocal microscope using a Plan Apochromat 100×/1.4 Oil DIC objective (Cell Observer Spinning Disk; Zeiss). For whole kidney section recording, images were acquired using Zeiss Mirax Midi slide scanner, stitched and analyzed using Pannoramic Viewer software.

### Electron microscopy

Kidneys were perfusion-fixed *in situ* as described^49^ using 0.1% (v/v) glutaraldehyde, then post-fixed therein overnight at 4 °C. Very small blocks were post-fixed with 1% (w/v) OsO_4_ in 0.1 M cacodylate buffer for 1 h, rinsed in veronal buffer (4 × 5 min) and stained overnight “en bloc” in 1% neutral uranyl acetate, all at 4 °C. Alternatively, samples were soaked in 2% glutaraldehyde containing 0.3% ruthenium-red to test for impermeability of junctions, as described^63^. After extensive washing in veronal buffer, blocks were dehydrated in graded ethanol and embedded in Spurr. Ultrathin (70 nm nominal) sections were obtained with a Reichert ultramicrotome (Reichert), collected on rhodanium 400 mesh grids and contrasted with 3% uranyl acetate followed by lead citrate, 10 min each. Grids were washed with water, dried, and examined in a FEI CM12 electron microscope operating at 80 kV.

### RT-qPCR

Total RNA was extracted (SV total RNA isolation system; Promega). Aliquots of 300 ng RNA were reverse-transcribed by M-MLV reverse transcriptase (Invitrogen) with random hexamers, as described^64^. Primer sequences used are described in Supplementary Table III. Real-time qPCR was performed as described^64^, in presence of 250 nM of specific primers with Kappa SYBR Fast qPCR Master Mix (Kapa Biosystems) on a CFX96 touch real-time PCR Detection System (Bio-Rad). Results are presented as Delta Ct values, normalized to HPRT-1, used as internal standard. Values of 0 indicate identical target mRNA levels and higher values indicate lower levels, assuming equal amplification efficiency.

### Western blotting

Kidneys were homogenized in lysis buffer containing 10 mM 3-[(3-cholamidopropyl)dimethylammonio]-1-propanesulfonate hydrate (CHAPS), 20 mM HEPES, pH 7.4, 150 mM NaCl, 2 mM CaCl_2_ with freshly added protease inhibitors (CompleteTM, Roche) as well as sodium orthovanadate, sodium pyrophosphate and sodium fluoride (2 mM each, Sigma-Aldrich). Kidneys were homogenized using a mortar-pestle and sonicated for 15 sec and lysates were centrifuged at 3000 rpm (Eppendorf centrifuge 5430 R) for 3 min at 4 °C and protein concentration in the supernatant was estimated by BCA. Proteins (20 µg protein/lane) were resolved into precasted gels (Mini protean TGX 4–15%, 456–1083, Bio Rad). For urines, sample volumes equivalent of 0.5 µg creatinine were resolved on precasted gels (Mini protean TGX 4–15%, 456–1083, Bio Rad). Antibodies are also listed in Supplementary Table II. Peroxidase-labelled secondary antibodies were revealed by enhanced chemiluminescence. Quantification was performed using imageJ software.

### Statistical analysis

Statistical significance was tested using Mann-Whitney test for single comparisons, or Tuckey test and Kolmogorov-Smirnov for multiple comparisons. Differences were considered significant if p < 0.05.

## Electronic supplementary material

Supplementary material
