# Supplementary material for: Vps34/PI3KC3 deletion in kidney proximal tubules impairs apical trafficking and blocks autophagic flux, causing a Fanconi-like syndrome and renal insufficiency
Source: Sci Rep. 2018 Sep 20;8:14133. doi: 10.1038/s41598-018-32389-z (PMC6148293; doi:10.1038/s41598-018-32389-z)
Supplement: Supplementary file 1 — Supplementary material [file 41598_2018_32389_MOESM1_ESM.pdf]

Vps34/PI3K3C3 deletion in kidney proximal tubules impairs apical trafficking and blocks autophagic flux, causing a Fanconi-like syndrome and renal insufficiency

Giuseppina Grieco[1], Virginie Janssens[1], Héloïse P. Gaide Chevronnay[1], Francisca N'Kuli[1], Patrick Van Der Smissen[1], Tongsong Wang[1], Jingdong Shan[2], Seppo Vainio[2], Benoit Bilanges[3], François Jouret[4], Bart Vanhaesebroeck[3], Christophe E. Pierreux[1*#], and Pierre J. Courtoy[1#].

*corresponding author
#equal senior authors

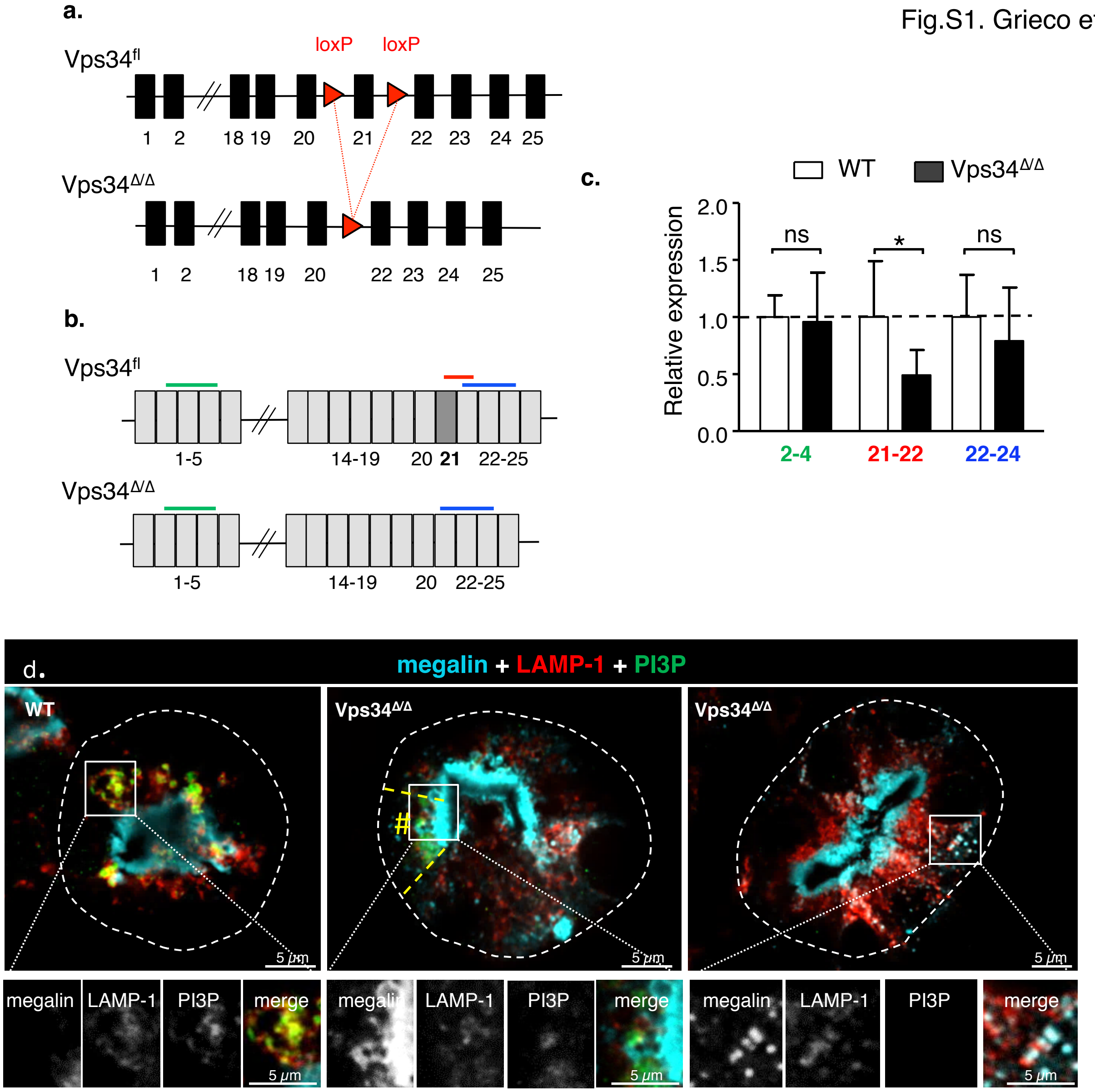

Supplementary Fig. S1. Validation of conditional Vps34 inactivation in PTCs. a. Genomic DNA. Vps34 allele showing loxP sites flanking exon 21 encoding the crucial Ala[730]-Thre[754] sequence in the PI3-kinase domain (above) and expected in frame deletion by Cre-recombinase (below). Conditional deletion in kidney PTCs was driven by Wnt4-Cre (mouse model A) or Pax8-Cre (mouse model B). ***b. Alignment of Vps34 amplicons*** probing mRNA expression upstream (green bar), downstream (blue bar) and over excised sequences (red bar; for primers, see Supplementary Table I). ***c. RT-qPCR*** of WT versus Vps34$^{\Delta/\Delta}$ (Pax8-Cre;Vps34$^{fl/fl}$) on P7 kidney lysates (means ± SD of 8 samples; *, $P<0.05$ by Mann-Whitney test). ***d. PI3P immunofluorescence in WT versus Pax8-Vps34$^{\Delta/\Delta}$ at P7.*** Broken white lines indicate PT contours. Broken yellow lines distinguish a PTC (#) with preserved restricted subapical localization of megalin. Boxed areas are enlarged below with single emissions images in white then merge in colors. PI3P (green) is compared to megalin (blue) and LAMP-1 (late endosomes/ lysosomes, red). In WT, PI3P is mostly detected below the subapical megalin layer, at the level of late endosomes/lysosomes. In Vps34$^{\Delta/\Delta}$, PI3P signal is strongly decreased in most but not all cells, compatible with mosaicism.

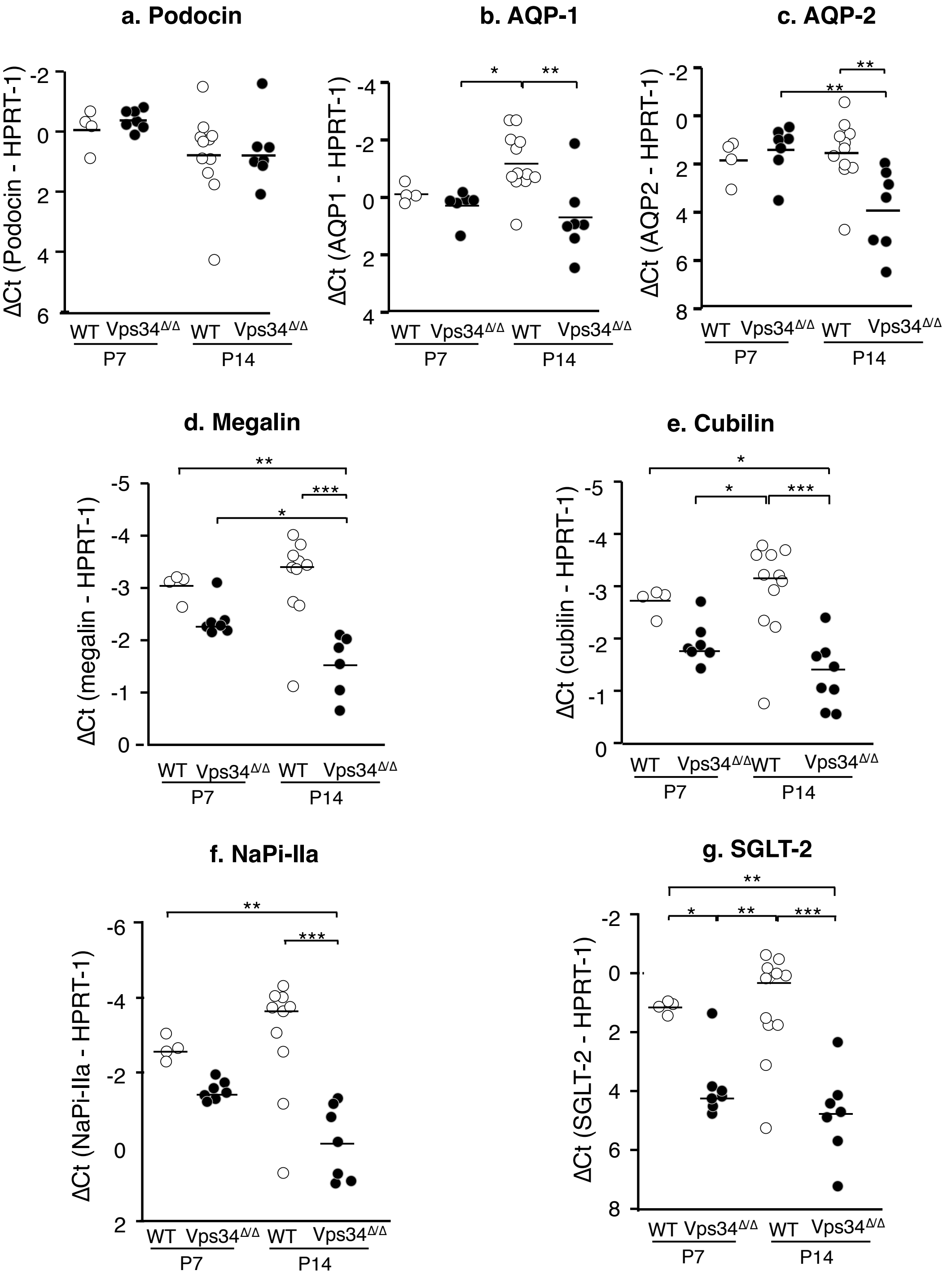

Supplementary Fig. S2 RT-qPCR. Expression in total kidney lysates from WT *vs* Vps34$^{\Delta/\Delta}$ pups at P7 and P14 of segmental nephron markers (glomeruli, podocin; proximal tubules, AQP-1; distal tubules, AQP-2), tandem endocytic receptors restricted to proximal tubules (megalin, cubilin) and major co-transporters of the proximal tubules (for phosphate, NaPi-IIa; for glucose, SGLT-2). Individual values are shown; bars denote medians. Statistical analysis by Tukey's multiple comparison test (non significant differences are not represented; *, p< 0.05; **, p<0.01; ***, p<0.001). Note preservation of podocin expression in Vps34$^{\Delta/\Delta}$ up to P14, contrasting with a decreased expression of all other constituents tested.

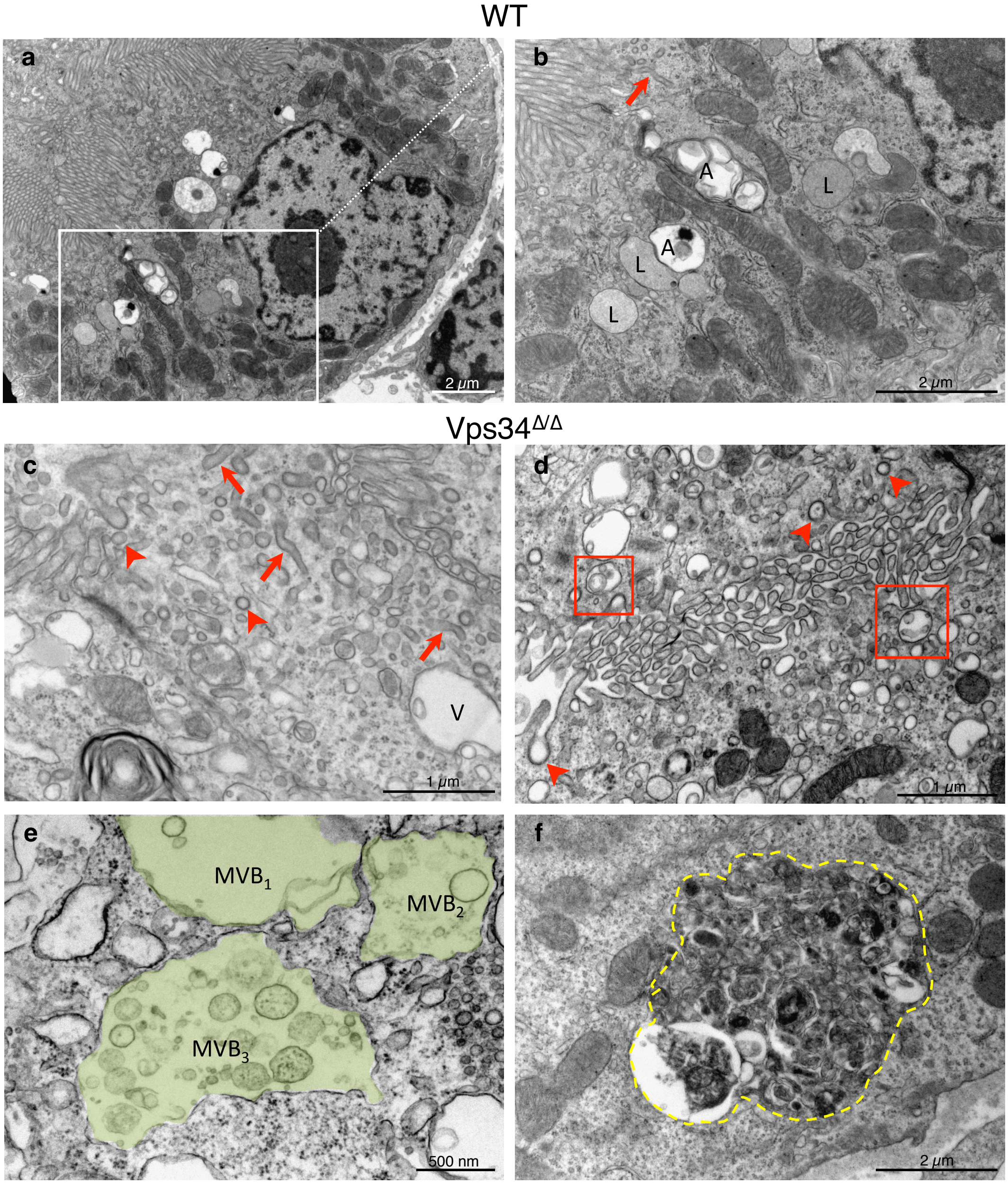

Supplementary Fig. S3. Conventional electron microscopy. Upper panel shows fully-differentiated WT PTCs at P7 (a) with magnification at the right (b). Note extensive brush border, numerous lysosomes with amorphous grey matrix (L) and two autophagosomes (A). Lower panel, representative views of Pax8-Vps34$^{\Delta/\Delta}$ PTCs at P7 (c, d) or P14 (e, f). Note the contrast between preserved PTCs in panel (c) showing a well-developed apical endocytic apparatus with numerous tubules *vs* altered PTCs at (d) with «disconnected» vesicles (no visible tubule) and vacuoles bearing intravesicular profiles (boxed in red); arrowheads, clathrin-coated profiles; arrows, dense apical (recycling) tubules visible only in (c); V, normal apical vacuole. In (e), enlarged multivesicular bodies without (MVB1) or with heterogenous content (MVB2,3). In (f), the yellow broken line delineates a huge lysosome with structured electron-dense content (residual body) but no mitochondrial remnant. Note mitochondrial integrity in this field. For junctions, see Supplementary Fig. S4 online.

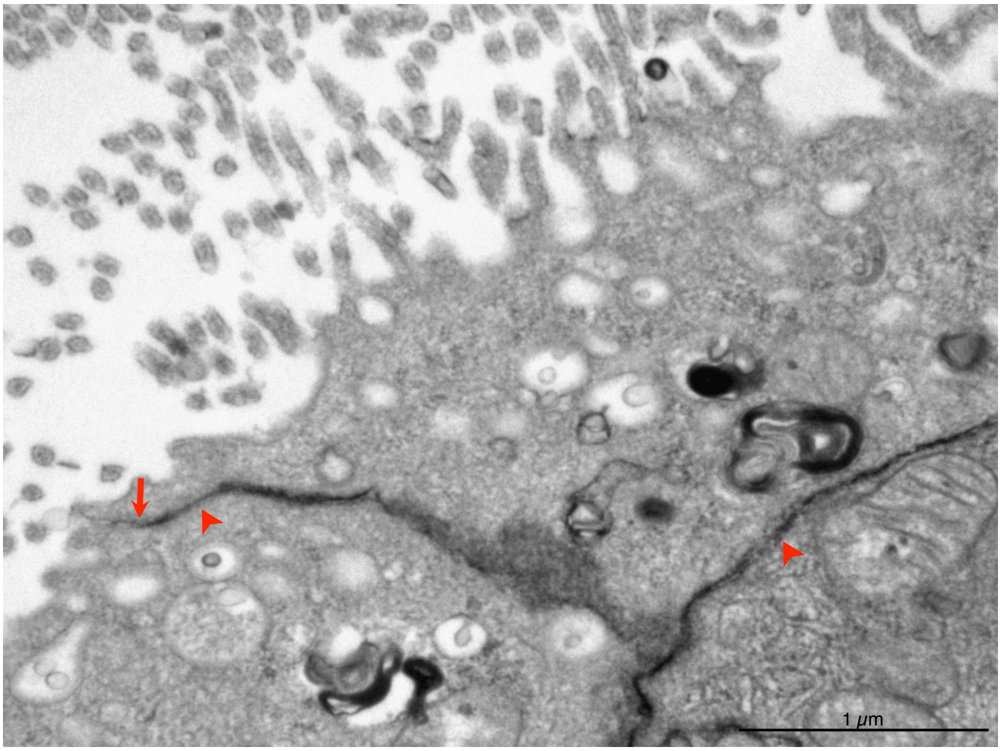

Supplementary Fig. S4. In situ assay of tight junction integrity by ultrastructural tracing of ruthenium red. Small pieces of Pax8-Vps34$^{\Delta/\Delta}$ kidney cortex at P7 were fixed by immersion in glutaraldehyde supplemented by the electron-dense tracer, ruthenium red (RR), diffusing from tissue interstitium. Arrowheads indicate continuous labeling of lateral membrane by diffusing RR. Arrow shows abrupt arrest of RR by tight junction, so that apical membrane is not labeled.-

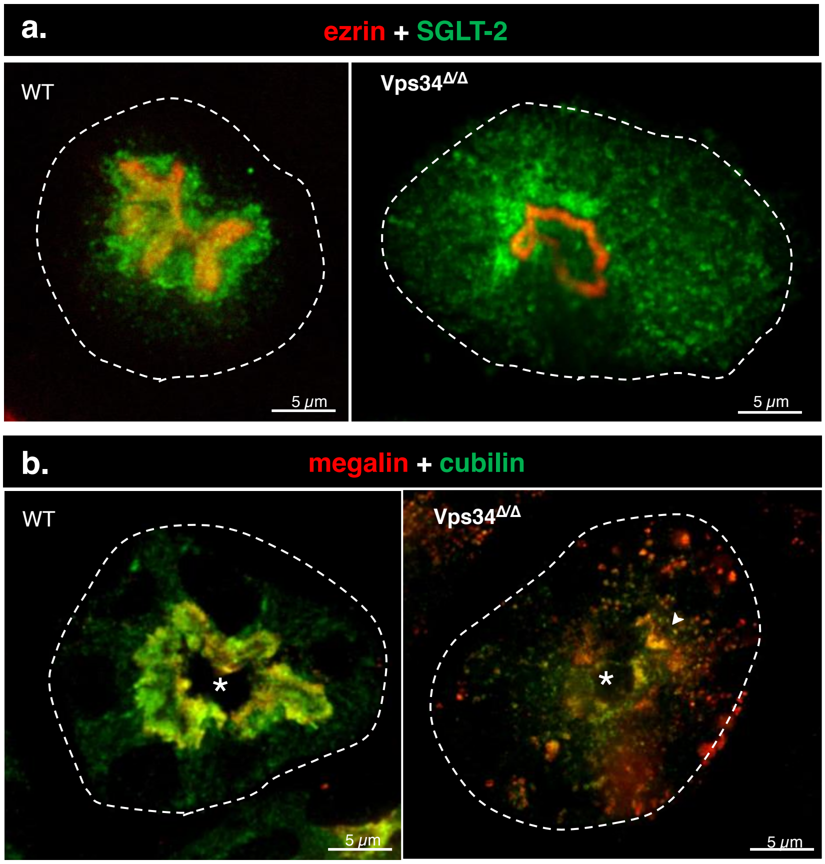

Supplementary Fig. S5. Mislocalization of SGLT-2 and cubilin in Pax8-Vps34$^{\Delta/\Delta}$ PTCs at P14 (confocal immunofluorescence). Asterisk denotes tubular lumen. Broken white lines indicate PT contours. Asterisks denotes tubular lumen. ***a.*** In WT PTCs, SGLT-2 (green) partially distributes at the brush border (ezrin, red; functional pool) and partially to the subapical layer. Vps34$^{\Delta/\Delta}$ causes extensive mislocalization to deep cytoplasmic dots while ezrin at brush border is preserved. ***b.*** In WT PTCs, cubilin (green) co-localizes with megalin (red) at the subapical layer (yellow signal) and similarly relocalizes in Vps34$^{\Delta/\Delta}$ to some cytoplasmic dots (arrowhead; fainter cubilin over megalin signal results from more severe decreased expression - see Supplementary Fig. S2 online).

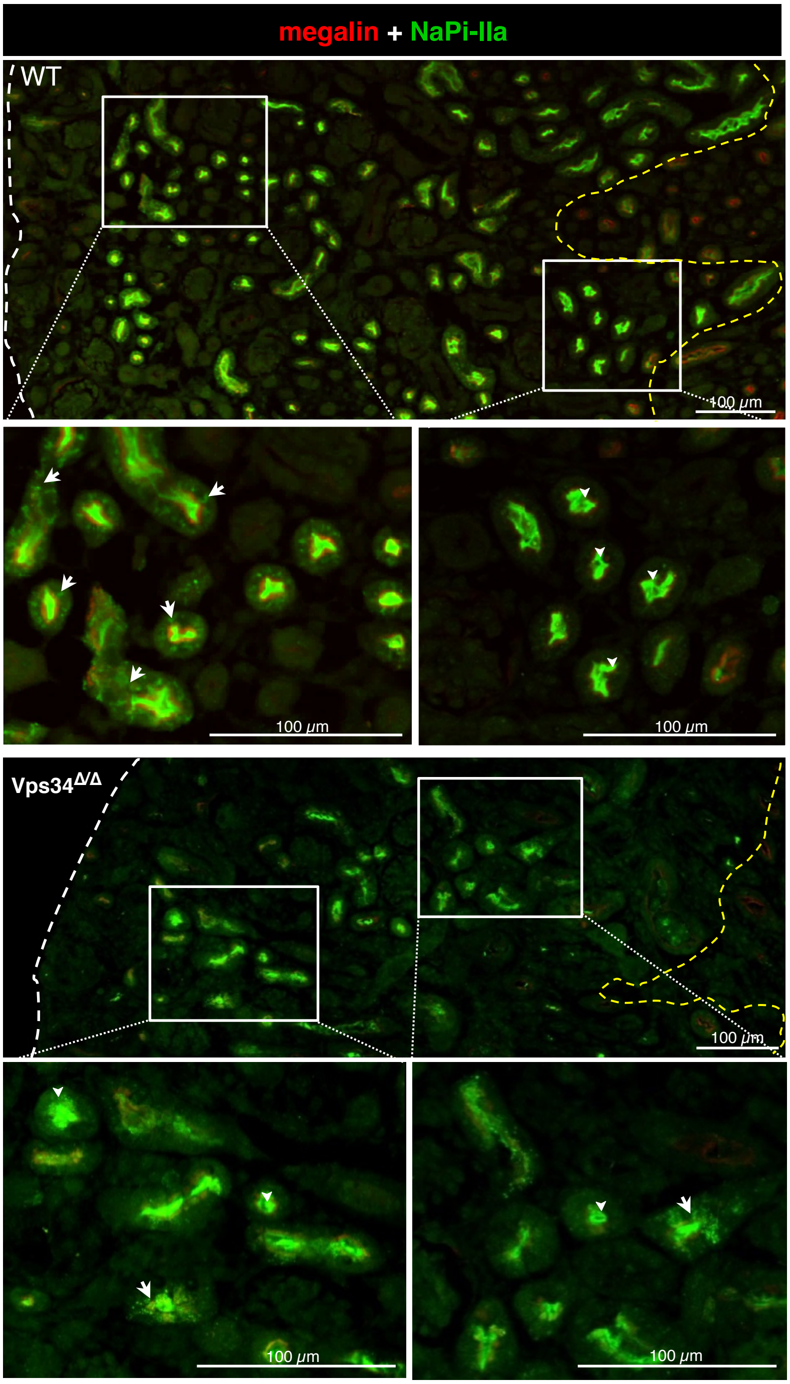

Supplementary Fig. S6. Low magnification view of cortical section at P5 in WT versus Wnt4-Vps34$^{\Delta/\Delta}$ after double immuno-fluorescence for NaPi-IIa (green) and megalin (red). Broken white lines at left denote kidney surface; broken yellow lines at right delineate cortico-medullary junction. Boxed areas are enlarged below. In WT kidney, NaPi-IIa expression is restricted to cortex; megalin also encompasses the outer stripe of outer medulla (right of dashed yellow line). Notice stratification in WT cortex between a superficial layer with immature NaPi-IIa pattern, i.e. combining apical brush border localization with perpendicular cytoplasmic stripes (arrows), and a deep layer characterized by mature pattern only (i.e. restricted to brush border, arrowheads). Vps34 $^{\Delta/\Delta}$ cortex is less developed and immature pattern is mixed with mature pattern in both superficial and deep cortex. Full-section images were acquired using a Zeiss Mirax Midi microscope. For quantification, see Supplementary Fig. S7 online.

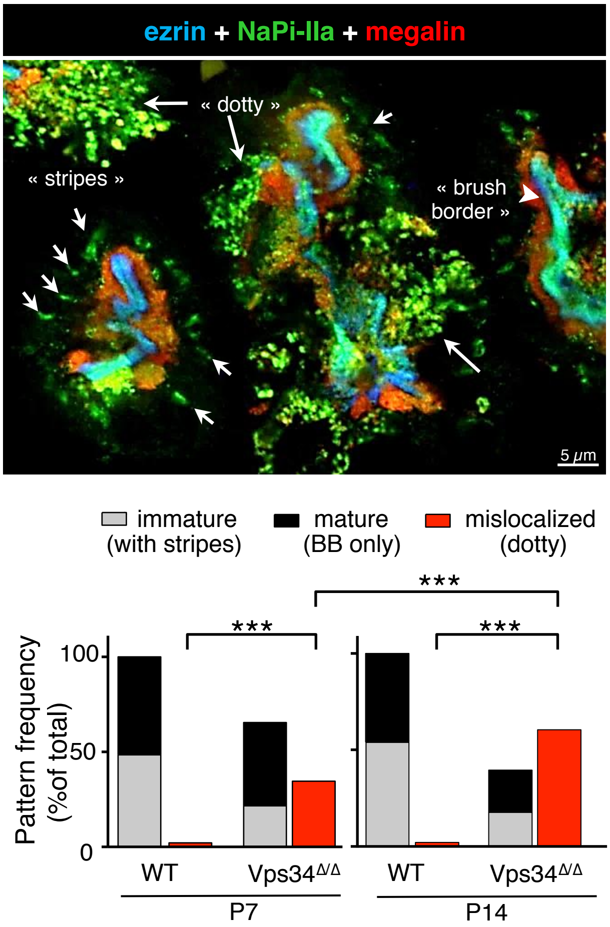

Supplementary Fig. S7. Quantification of NaPi-IIa redistribution in Pax8-Vps34$^{Δ/Δ}$PTCs by confocal microscopy. The upper panel illustrates three typical confocal imaging patterns; frequency is quantified in lower panel. (i) immature (mixed distribution at brush border and deeper lateral stripes, short arrows); (ii) mature/differentiated (only brush border, arrowhead at right); (iii) redistributed (mostly or completely as cytoplasmic dots, long arrows). Bar histograms are means ± SD of 752 to 1040 individual PTCs sectioned across cell nucleus per condition, each pooled from 120 fields of 5363 μm^2 per kidney cortex of 3 mice. Disrupted tubules (at P14) were excluded from the analysis. ***, P<0.001 by Kolmogorov-Smirnov test.

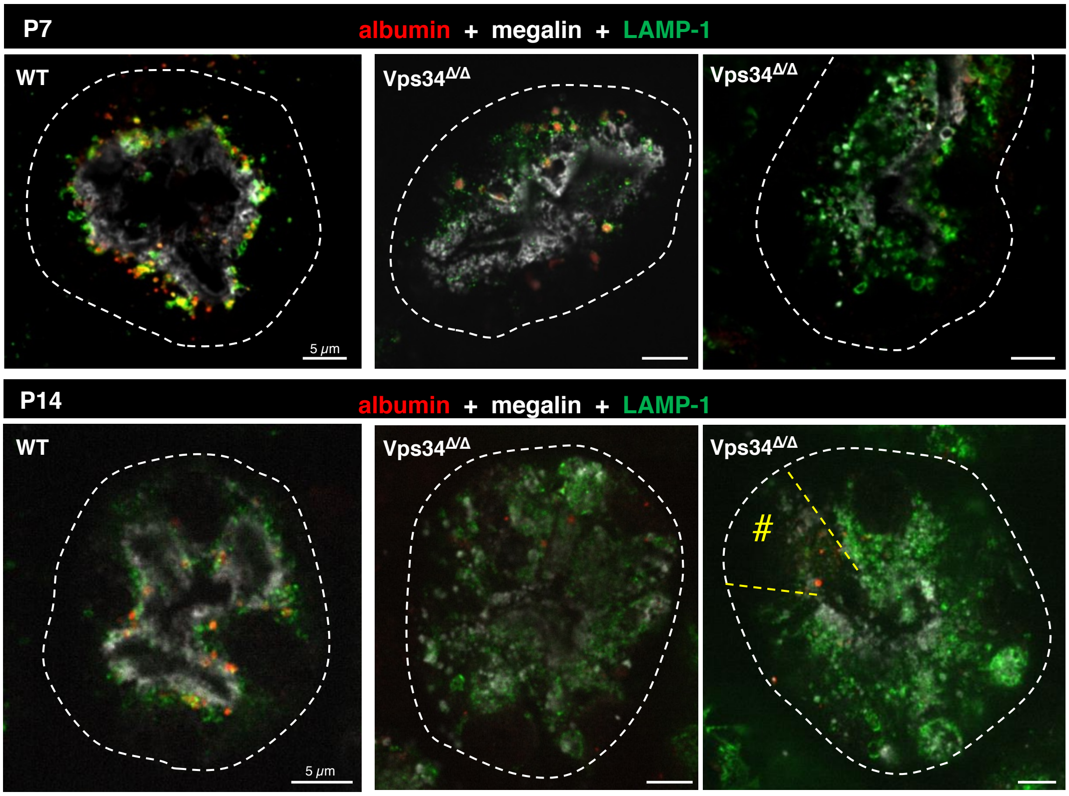

Supplementary Fig. S8. Vps34$^{\Delta/\Delta}$ PTCs show much reduced albumin steady-state content. Kidney sections from WT and Vps34 $^{\Delta/\Delta}$ pups at P7 (upper panel) and P14 (lower panel) were triple-immunolabeled for albumin as endocytic cargo marker (red), megalin as endocytic receptor (white) and LAMP-1 as membrane marker for lysosomes into which albumin is transiently sequestered before undergoing degradation. Broken white lines delineate PT contours. The pair of radial broken yellow lines defines an unusual PTC with normal appearance in Vps34$^{\Delta/\Delta}$ mice, attributed to mosaicism (#, notice normal abundance and apical localization of LAMP-1 organelles). In WT PTCs at P7 and P14, albumin signal appears as apical dots, largely co-localizing with LAMP-1 below the continuous megalin layer. Red-only dots presumably correspond to endosomes. In Vps34$^{\Delta/\Delta}$ PTCs, progression of alterations is suggested from central panels to right panels. Notice the severe reduction of albumin signal especially at P14.

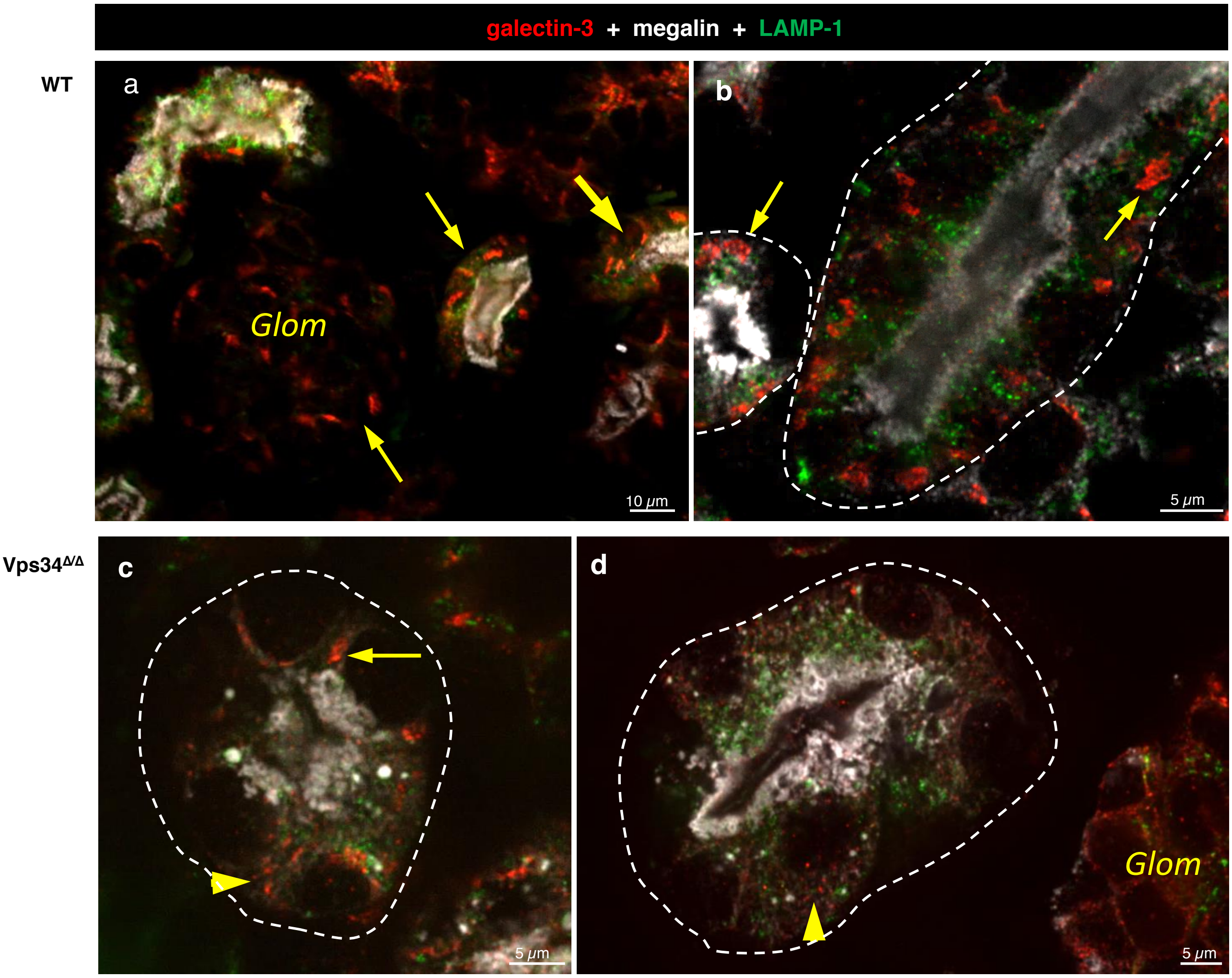

Supplementary Fig. S9. Vps34 deletion in PTCs disrupts the galectin-3-labeled perinuclear compartment. Kidney sections from WT and Vps34 $^{Δ/Δ}$ mice at P14 were triple-immunolabeled for galectin-3 (red), megalin (white) and LAMP-1 (green). Broken white lines delineate PT contours; *Glom*, glomerulus. In WT kidney, galectin-3 antibodies specifically label a rubbon in each cell (arrows), usually in a perinuclear position (thick arrow). In Vps34 $^{Δ/Δ}$ PTCs, galectin-3 signal is usually dispersed into multiple dots (arrowheads), suggesting early dispersion of the perinuclear organelle. Notice absence of co-localization between galectin-3 dots and LAMP-1 dots, better visible as individual organelles at P7 than at P14, due to LAMP-1 overexpression at this stage.

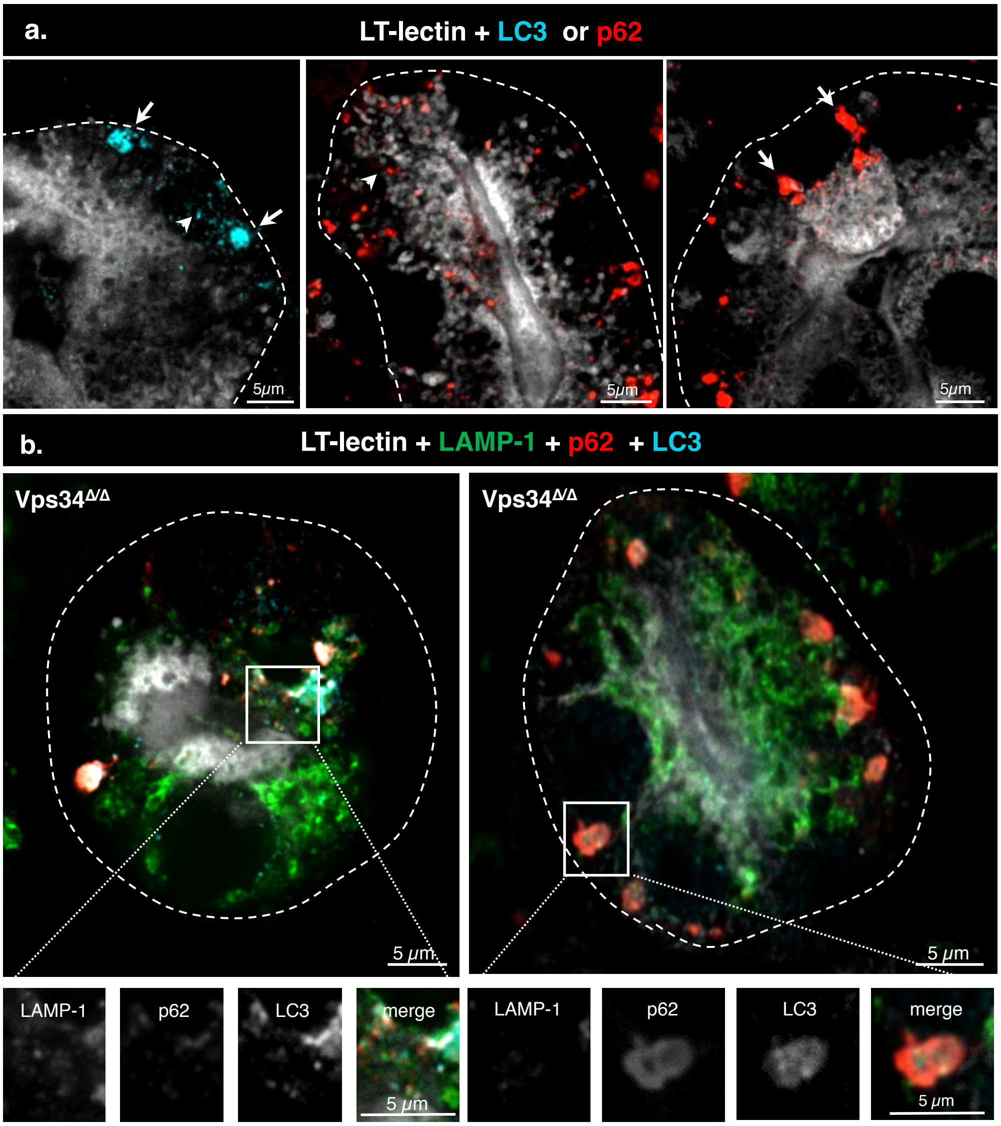

Supplementary Fig. S10. Progression of autophagic punctae. a. Specificity of immunolabelling. Vps34$^{\Delta/\Delta}$ kidneys were incubated with either rabbit anti-LC3 (blue) or guinea pig anti-p62 (red) alone, as indicated, then incubated with a combination of the two appropriate secondary antibodies and lotus-tetragonolobus lectin (LT-lectin, white) to identify PTCs and the integrity of their subapical compartment. Notice absence of cross-reactivity between LC3 and p62 immunofluorescence. In the left panel, notice many small LC3-punctae, most abundant in the apical cytoplasm (arrowheads), and only two larger LC3-punctae above the basal membrane, indicative of early alteration. Central and right panels show respectively mostly small scattered p62-punctae (early alteration) and several larger basal p62-punctae (indicative of more advanced alteration). ***b. Segregation from late endosomes/lysosomes***. Quadruple labeling for LT-lectin (white), LAMP-1 (green), LC3 (cyan) and p62 (red) in Vps34$^{\Delta/\Delta}$ kidneys (left panel, early alterations; right panel, advanced alterations). Boxed areas are enlarged below. Notice early apical small LC3 punctae then large basal assemblies double-labeled for p62 and LC3, but fully segregated from LAMP-1.

Fig.S11 Grieco et al
(Full-length blots shown in Fig 2b)

A) P7

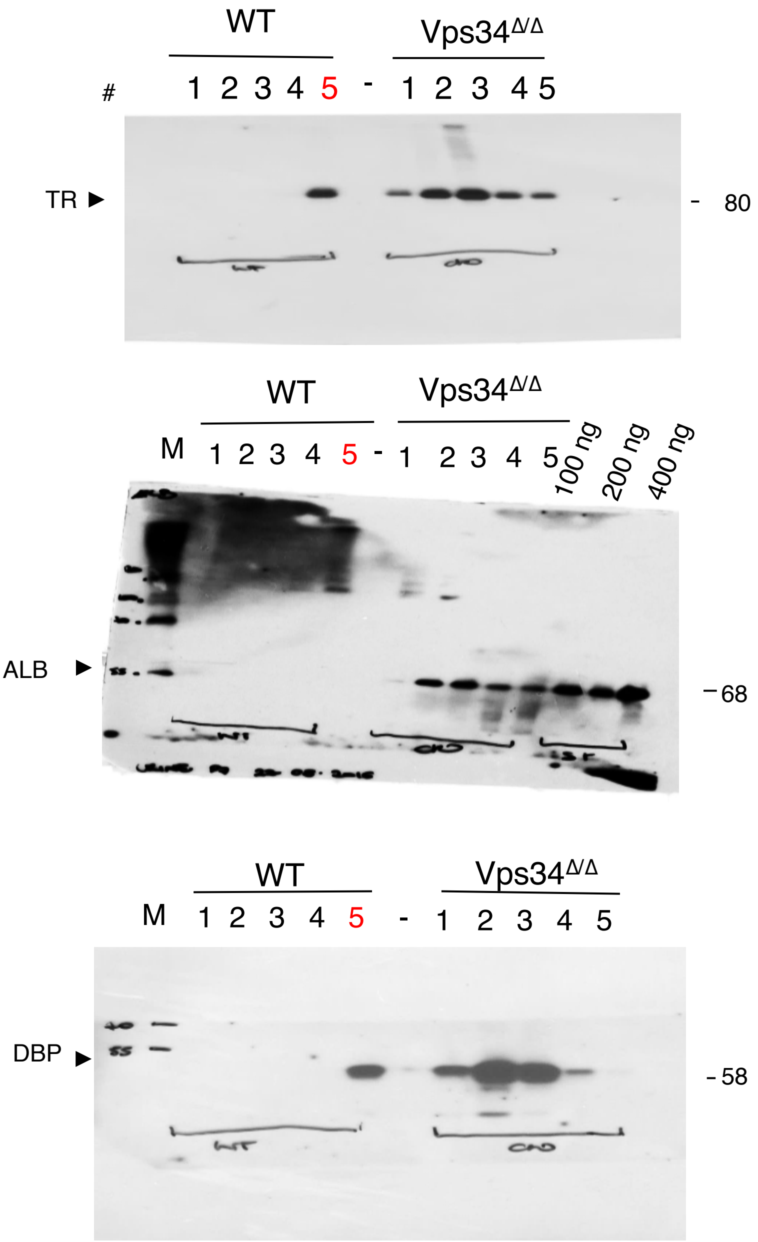

Please note:

- Sample #5 in P7 had been unfortunately misclassified as a WT and this lane has thus been removed in silico. Fig 2b shows comparison at identical blot exposure of verified WT and cKO samples.
- Three additional lanes at right on the central gel were loaded with the indicated increasing amounts of albumin. These are not shown in the blots shown at Fig. 2b left, but were used to quantify the IgG/albumin ratio shown at Fig2b right.

Fig.S11 Grieco et al
(Full-length blots shown in Fig 2b)

B) P14

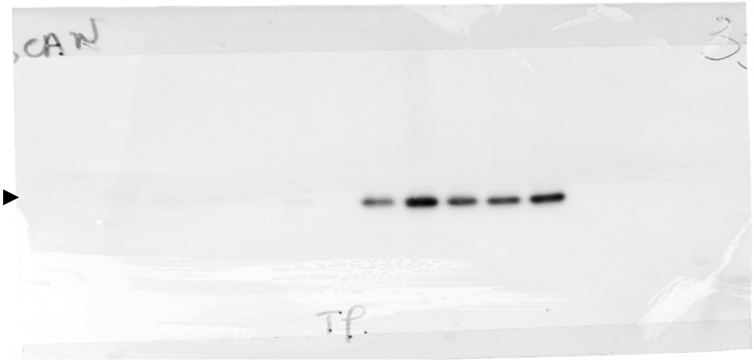

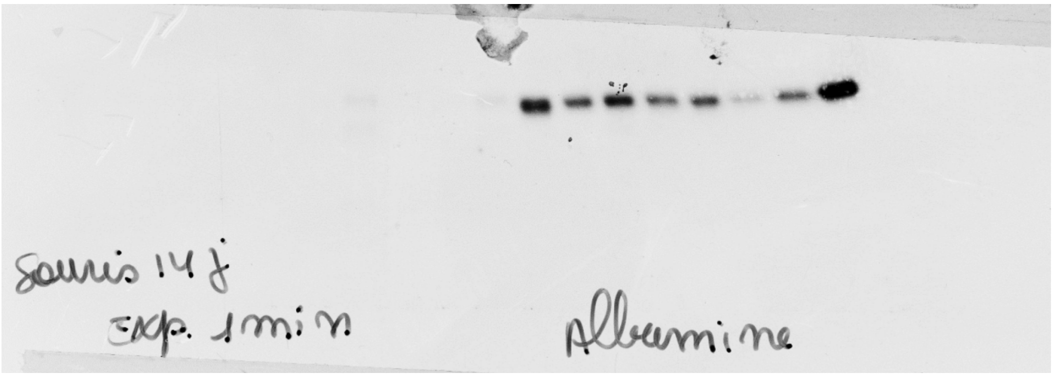

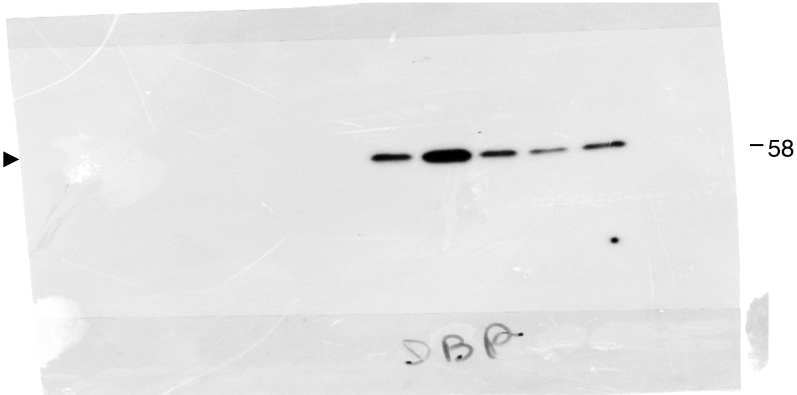

Supplementary Table I : Primers

gene	forward primer (5'-->3')	reverse primer (5'-->3')
Vps34 2-4	TGGAATTGGAACGAGTGGCT	CACGTTAGGCCAGACCTTCA
Vps34 21-22	TTGGAGTTGGAGACCGGCA	TCCTTGTTCAGCTTCATCGGAG
Vps34 22-24	GATGGTGGAAGGGATGGGTG	CGCTCTCGTCGATCAGACTC
podocin	AAGGACAGATATGGGCACTGTCA	CCAGGAGCACCTAAGCTATGGAA
AQP-1	GCTGTCATGTACATCATCGCCCAG	AGGTCATTGCGGCCAAGTGAAT
AQP-2	TGAGCCTCAAGAAGGGTCTC	TCTCCAGAGCTCTCCGTCTC
megalin	CAGTGGATTGGGTAGCAGGA	GCTTGGGGTCAACAACGATA
cubilin	TCATTGGCCTCAGACATTCC	CCCAGACCTTCACAAAGCTG
NaPi-IIa	AGGTGAGCTCCGCCATTCCGA	CCCTGCAAAAGCCCGCCTGA
SGLT-2	GCTGGTCATTGGTGTTGGCTTGTG	GAACAGAGAGGCTCCAACCGGC
HPRT-1	ACATTGTGGCCCTCTGTGTG	TTATGTCCCCCGTTGACTGA

Supplementary Table II: Antibodies and use

Reagents	Source	Reference	Application	IF dilution	WB dilution
rabbit MAb anti-Vps34/PI3K-III	Abcam	ab124905	frozen sections	1/100	
mouse MAb anti-ezrin	Thermo Scientific	MS-661-P1	paraffine/frozen sections	1/200	
rabbit anti-mouse NaPi-IIa	Dr. J. Biber	Custer at al. 1994	paraffine/frozen sections	1/1000	
sheep anti-rat megalin	Dr. P. Verroust	Verroust et al. 2002	paraffine/frozen sections	1/1000	1/5000
rabbit anti-human transferrin	Dako	A0061			1/1000
goat anti-mouse albumin	Santa Cruz	sc-46293			1/1000
rabbit anti-human DBP	Dako	A0021			1/1000
rabbit anti-human Albumin	Dako	A0001	paraffine sections	1/1000	
rabbit anti-NHERF-1	Dr. C. Yun	N/A	paraffine/frozen sections	1/1000	
mouse MAb anti-Na^+ /K^+-ATPase	Hybridoma Bank	a6F	frozen sections	1/400	
rat MAb anti-LAMP-1	Hybridoma Bank	1D4B	paraffine/frozen sections	1/100	
rabbit anti-LAMP-2A	Abcam	ab18528	frozen sections	1/200	
mouse MAb anti-PI(3)P*	Echelon	Z-P003	paraffine sections	1/400	
rabbit anti-rat cubilin	Dr. R. Kozyraki	Verroust et al. 2002	frozen sections	1/1000	1/5000
rabbit anti-human SGLT-2	Santa Cruz	sc-98975	frozen sections	1/100	
rabbit MAb anti-Tom20	Cell Signaling	42406	paraffine/frozen sections	1/200	
guinea pig anti-p62	ARP	03-GP62C-	paraffine/frozen sections	1/400	
rabbit MAb anti-LC3B	Cell Signaling	3868	frozen sections	1/200	
rabbit MAb anti-Galectin-3	Abcam	ab76245	paraffine sections	1/250	
mouse anti-Rab11	DB Biosciences	610656	paraffine sections	1/500	
TexasREd-ovalbumin	Invitrogen	O23021	frozen sections	180µg	

*Anti-PI3P antibodies were cleared of aggregates by ultracentrifugation in a Ti50 rotor (Beckman) at 40,000 rpm for 1 h (6 10^6 *g* xmin).
